# The microbiome of *Riccia* liverworts is an important reservoir for microbial diversity in temporary agricultural crusts

**DOI:** 10.1186/s40793-023-00501-0

**Published:** 2023-06-01

**Authors:** Wisnu Adi Wicaksono, Bettina Semler, Martina Pöltl, Christian Berg, Gabriele Berg, Tomislav Cernava

**Affiliations:** 1 Institute of Environmental Biotechnology, Graz University of Technology, Graz, 8010 Austria; 2 Institute of Biology, University of Graz, Graz, 8010 Austria; 3 Leibniz Institute for Agricultural Engineering and Bioeconomy (ATB), Potsdam, Germany; 4 Institute for Biochemistry and Biology, University of Potsdam, Potsdam, Germany; 5 Graz University of Technology, Graz, Austria

**Keywords:** Bacteria, Fungi, Methylobacteria, Liverworts, Microbiome, Crop history

## Abstract

**Background:**

The microbiota of liverworts provides an interesting model for plant symbioses; however, their microbiome assembly is not yet understood. Here, we assessed specific factors that shape microbial communities associated with *Riccia* temporary agricultural crusts in harvested fields by investigating bacterial, fungal and archaeal communities in thalli and adhering soil from different field sites in Styria and Burgenland, Austria combining qPCR analyses, amplicon sequencing and advanced microscopy.

**Results:**

*Riccia spec. div.* was colonized by a very high abundance of bacteria (10^10^ 16S rRNA gene copies per g of thallus) as well as archaea and fungi (10^8^ ITS copies per g of thallus). Each *Riccia* thallus contain approx. 1000 prokaryotic and fungal ASVs. The field type was the main driver for the enrichment of fungal taxa, likely due to an imprint on soil microbiomes by the cultivated crop plants. This was shown by a higher fungal richness and different fungal community compositions comparing liverwort samples collected from pumpkin fields, with those from corn fields. In contrast, bacterial communities linked to liverworts are highly specialized and the soil attached to them is not a significant source of these bacteria. Specifically, enriched *Cyanobacteria, Bacteroidetes* and *Methylobacteria* suggest a symbiotic interaction. Intriguingly, compared to the surrounding soil, the thallus samples were shown to enrich several well-known bacterial and fungal phytopathogens indicating an undescribed role of liverworts as potential reservoirs of crop pathogens.

**Conclusions:**

Our results provide evidence that a stable bacterial community but varying fungal communities are colonizing liverwort thalli. Post-harvest, temporary agricultural biocrusts are important reservoirs for microbial biodiversity but they have to be considered as potential reservoirs for pathogens as well.

**Supplementary Information:**

The online version contains supplementary material available at 10.1186/s40793-023-00501-0.

## Introduction

The plant microbiome can fulfill important functions for the health and growth its host. A wide range of microorganisms are living either in a symbiotic, commensal, pathogenic, essential or neutral relationship with land plants [1, 2], and they are involved in major functions like plant nutrition and plant resistance to biotic and abiotic stress [3]. During 450 million years of colonization and diversification on land, plants and microbes have co-evolved [4]. This is reflected in the phylosymbiosis concept [5], which was especially studied for many crops but less for bryophytes [6].

Liverworts belong, just like mosses and hornworts, to the group of bryophytes which play an important role in nutrient and carbon cycles, plant biomass and community maintenance, and biodiversity [7, 8]. Recently, it was described that *Sphagnum* mosses are characterized by a more ancient mechanism of plant microbiota assembly [9]. Liverworts are living in association with fungi before the true mycorrhiza evolved, and interestingly, fungi originally switched from the gametophytes of liverworts to the roots of the sporophytes of tracheophytes and not vice versa [10]. There are only a few genera of thallose liverworts like *Riccia*, which have no fungal symbionts. Members of the genus *Riccia* form biological crusts in their native environments, and some species are unique because they adapted to traditional agricultural practices. They form temporary biocrusts in the period after harvest and before sowing, which ensures the maintenance of a soil-plant continuum under agricultural conditions. This is of high importance because agricultural biocrusts increase soil carbon sequestration and reduce greenhouse gas emissions [11]. An expert panel from 195 countries recently suggested to increase global soil organic carbon stocks by 0.4% per year to compensate for greenhouse gas emissions, the ‘4 per 1000’ agreement. In this context, the application of biocrusts is promising but their microbiome constituents, which may support them in these roles, are only sparsely understood [11]. Therefore, in this study, we focused on the analysis of the specialized microbiome of *Riccia* biocrusts.

Previous studies have provided first evidence that liverworts are colonized by specific bacterial communities [12]. The obtained results indicated that methylobacteria might be a stable component of the liverwort microbiome. Pink-pigmented methylobacteria are methylotrophic bacteria that live on plant surfaces and exhibit versatile traits like the ability to scavenge for a wide variety of carbon sources which has allowed them to specifically adapt to the phyllosphere environment [13–16]. This bacterial group can also contribute to overall plant health [17, 18]. In the present study, we aimed to deepen our understanding related to the specificity of the liverwort microbiome, with a focus on bacterial as well as fungal communities. We have implemented thallose liverworts for this approach, more precisely the two closely related species *Riccia glauca* L. and *Riccia bifurca* Hoffm. The detailed aims of this study were to analyze the effects of field type (geography and cropping history) on the microbiome of the selected liverwort species. We hypothesized that liverworts accumulate specific microorganisms compared to the surrounding soil, and that they would harbor a common bacterial and fungal core microbiome that is present in all samples. In addition, adhering soil samples were analyzed in order to identify potential cues for microbiome assembly in liverworts and we looked into their potential role as reservoir for plant pathogens.

## Materials and methods

### Sample collection and preparation

**Fig. 1:**
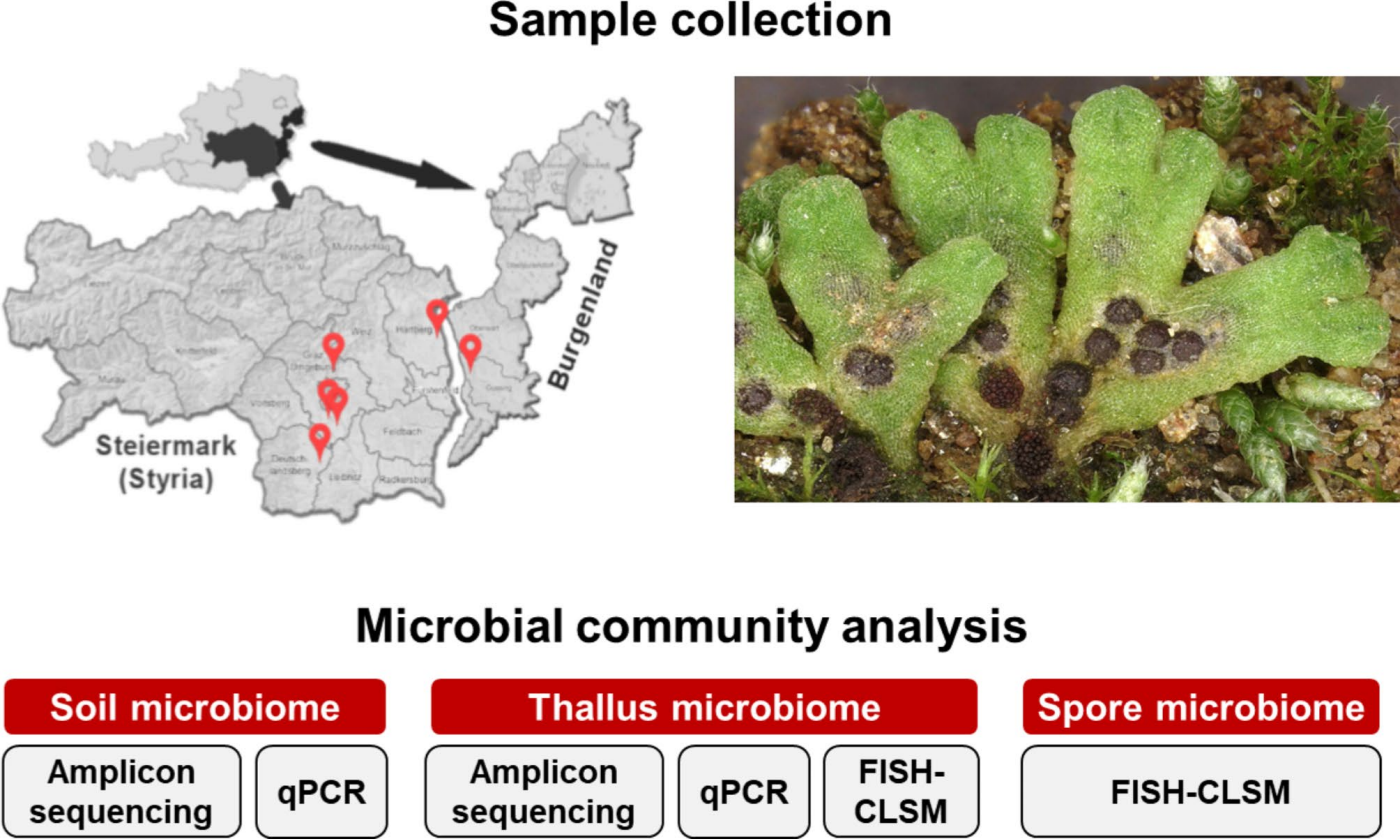
Overview of sampling locations and the conducted microbial community analyses

Liverwort samples were collected from corn fields and pumpkin fields, partially after harvest, from six different locations in Styria and Burgenland (Austria, Fig. 1). The collected specimens were identified as *Riccia glauca* L. and *Riccia bifurca* Hoffm (Supplementary Figure S1). A detailed sample description is provided in Supplementary Table S1. From each location, six different liverwort thalli were collected. After arrival in the laboratory, the liverwort thalli and the adhering soil were separated with sterile razor and forceps and subsequently washed with sterile water. The washing step was included to remove soil derived bacteria from the thallus surfaces. The samples were stored at -20 °C in sterile 2-ml reaction tubes until DNA extraction.

### Microscopic in situ visualization of bacterial colonization in spore and thallus of liverworts

Spore and thallus samples were fixed overnight in 4% paraformaldehyde/phosphate-buffered saline at 4 °C before FISH, as described previously [19]. To stain overall bacterial colonization and specific colonization of *Alphaproteobacteria* and *Gammaproteobacteria*, specific probes were implemented, including Cy3-labeled EUB338MIX [20, 21], ATTO488-labeled ALF968 [19] and Cy5-labeled GAM42a [22]. Calcofluor White (Sigma-Aldrich, Missouri, United States) was used to stain the host cell membranes in FISH samples. The bacterial colonization in the spore and thallus samples was visualized using a Leica TCS SPE confocal laser scanning microscope (Leica Microsystems, Mannheim, Germany). The confocal stacks were merged for all experiments to obtain a maximum projection of all channels.

### Total Community DNA extraction from liverwort samples

Each sample was processed with a DNA extraction kit (Fast DNA SPIN Kit for soil, MP Biomedicals, Solon, OH, United States) to extract the total community DNA from the liverwort thalli and the adhering soil. All total community DNA extractions were performed according to the manufacturer’s protocol. The quality and quantity of extracted DNA were evaluated with a NanoDrop 2000 device (Thermo Fisher Scientific; Wilmington, DE, United States). The extracted DNA samples were stored at -20 °C until further processing.

### Quantitative real-time PCR

Quantification of prokaryotic, archaeal, methylobacterial and fungal marker genes was done by quantitative PCR (qPCR) with total community DNA extracts from the samples. The molecular quantification was performed using the Rotor-Gene-6000 Real-Time Rotary Analyzer (Corbett Research, Sydney, Australia). Each sample was analyzed with three technical replicates. Prokaryotic 16S rRNA gene fragments were amplified with the primers 515f (5’-GTGYCAGCMGCCGCGGTAA-3’) and 806r (5’-GGACTACHVGGGTWTCTAAT-3’) [23]. For archaeal quantification, the primer pair 344aF (5´ - CGGGGYGCASCAGGCGCGAA − 3`) and 517uR (5´ - GWATTACCGCGGCKGCTG − 3`) was used [24]. For methylobacterial quantification the primers MxaFqPCRAF (5’ - CGTCAACGTCATGATGCT(C/G)T − 3’) and MxaFqPCRAR (5’ -GATGTCCTTGGCGAG(A/G)TG -3’) were used [25]. Finally, the primer pair ITS1f (5’-CTTGGTCATTTAGAGGAAGTAA-3’) and ITS2r (5’-TGTGTTCTTCATCGATG-3’) was used to quantify fungal abundance [26]. The standard curves were obtained using *in-house* isolates, i.e., *Pseudomonas brassicacearum* L13-6-12 (prokaryotic 16S rRNA gene), *Haloferax denitrificans* (archaeal 16S rRNA gene), *Methylobacterium mesophilicum* SAB-1 (methylobacterial mxa*F* gene), and *Lobaria pulmonaria* (fungal ITS gene). The standard regression curve (serial 1:10 dilutions) was employed to determine the gene copy numbers in the analyzed samples. These numbers were subsequently normalized according to the weight of initial sample. To calculate the bacterial abundance, we subtracted archaeal abundance from the abundance obtained for all prokaryotes.

### 16S rRNA and ITS metabarcoding and bioinformatic analysis

The V4 region of prokaryotic 16S rRNA genes was amplified with the primers 515f (5’-GTGYCAGCMGCCGCGGTAA-3’) and 806r (5’-GGACTACHVGGGTWTCTAAT-3’) [23]. The fungal ITS1 region was amplified with the primers ITS1f (5’-CTTGGTCATTTAGAGGAAGTAA-3’) and ITS2r (5’-TGTGTTCTTCATCGATG-3’) [26]. Each primer contained sample-specific barcodes for demultiplexing following the guideline of the Earth Microbiome Project protocol [27]. The following PCR program (96 °C 5 min to denature the DNA, 30 cycles at 96 °C for 60 s, 78 °C for 5 s, 54 °C for 60 s, 74 °C for 60 s, and 10 min at 72 °C for a final extension) was used to amplify 16S rRNA gene fragments. Specific peptide nucleic acid (PNA) oligomers were added to the PCR mix to prevent the amplification of mitochondrial (mPNA) or plastidial (pPNA) RNA genes from plant origin. For amplification of ITS genes, the following PCR program (96 °C for 5 min to denature the DNA, 30 cycles at 96 °C for 60 s, 58 °C for 60 s, 74 °C for 60 s, and 10 min at 74 °C for a final extension) was used. Purification of 16S rRNA and ITS amplicons were done using the Wizard SV Gel and PCR Clean-Up System (Promega, Madison, WI). The sequencing of the barcoded amplicons was performed on an Illumina MiSeq instrument (2 × 250 bp paired-end reads) by the sequencing provider Eurofins Genomics Europe Sequencing GmbH (Constance, Germany). Amplicon sequences were deposited at the European Nucleotide Archive (ENA) under the project number PRJEB52587.

Paired-end raw reads were demultiplexed and primer sequences were subsequently removed using cutadapt [28]. The demultiplexed reads were then imported to the open-source QIIME2 pipeline [29]. The DADA2 algorithm [30] was used to quality filter, denoise and remove chimeric sequences. This process resulted in representative sequences, called amplicon sequences variants (ASVs), and a feature table. ASVs were further classified using the vsearch algorithm against the SILVA v132 database for bacterial reads or UNITE database for fungal reads [31–33]. After filtering chimeric and non-prokaryotic reads (unassigned, mitochondrial and plastid sequences), the prokaryotic feature table contained 4,843,370 sequences. A total number of 21,496 ASVs remained in the dataset. The overall fungal community, assessed by ITS gene fragment amplicon sequencing, contained 2,603,314 sequences and these sequences were assigned to 3350 fungal ASVs. The amplicon sequencing datasets were normalized by randomly selecting subsets of sequences (6789 sequences for the prokaryotic dataset and 2498 sequences for the fungal dataset). We used the SourceTracker2 software [34] to estimate the microbiota proportion in thalli that originated from the soil. A deepening analysis was conducted to analyze the core microbiome, which was defined as ASVs with an occurrence in ≥ 90% of the total samples. To perform phylogenetic analyses, sequences of ASVs that were defined as members of the core microbiome were aligned using MUSCLE [35] and the distance matrices were calculated with the maximum-likelihood algorithm in MEGA X (Molecular Evolutionary Genetic Analysis [36]).

### Isolation and identification of Riccia-associated bacteria

The liverwort thalli were washed with sterile water to remove adhering soil. Approx. 0.1 g of liverwort thalli were cut into smaller fragments in 500 µL of 0.9% NaCl with a sterile scalpel. The samples were tenfold serially diluted in 0.9% NaCl and the resulting suspensions were then plated on King’s B (KB) medium routinely used for the isolation of plant-associated bacteria [37].

The agar plates were incubated at 25 °C in the dark and checked for bacterial growth every 2–3 days for a period of 2 weeks. We selected representative bacteria based on visual differences of bacterial colonies and their morphology (shape and color).

In total, 30 selected bacterial isolates were purified and subsequently identified by Sanger sequencing of their 16S rRNA gene segments using the universal bacterial primer pair 2 F and 1193R. The Sanger sequencing was conducted at LGC genomics (Berlin, Germany). The raw sequencing reads were quality filtered using BioEdit [38] to remove ambiguous sequences. The quality-filtered sequences were compared against NCBI standard database (nr/nt) using the Basic Local Alignment Search Tool (BLAST [39]).

### Statistical analysis

Statistical analysis and visualization of graphs were conducted in R studio v. 2021.09.0 [40, 41] unless stated otherwise. To evaluate significant differences (P < 0.05) in microbial gene copy numbers, the Kruskal Wallis test was used, followed by post-hoc Dunn’s tests for multiple pairwise comparisons. The ASV tables and taxonomic classifications were used as the input dataset for the microbial community analysis, which was performed using the phyloseq R package and MicrobiomeAnalyst [42, 43]. Furthermore, the dataset was rarefied by randomly selecting subsets of sequences with the fewest read counts. Plot bars were used to depict taxonomic composition. The Kruskal Wallis test, followed by post-hoc Dunn’s tests for multiple pairwise comparisons at *P* < 0.05, was used to identify changes in alpha diversity according to the Shannon diversity index (H’) using the rarefied dataset. The rarefied dataset was further used to generate non-metric Bray-Curtis dissimilarity matrices, which were then submitted to to a Permutational Multivariate Analysis of variance (Adonis, 999 permutations) to assess whether tested factors (field type and microhabitat, i.e., soil and thallus) had any significant effects on microbial community structures. A two-dimensional non-metric multidimensional scaling (NMDS) plot was generated to visualize the distance matrices. Additionally, the Bray Curtis dissimilarity matrix was also implemented to generate hierarchical clustering. Furthermore, calculation of linear discriminant analysis effect size (LEfSe) [44] was performed to identify taxa that were significantly enriched *P*_*adjusted*_ value was less than 0.05 in the thallus samples in comparison to the surrounding soil samples from each location. Sequences of ASVs that were enriched in thallus samples were compared against those of known origin using Eztaxon (www.ezbiocloud.net) and UNITE for bacterial and fungal ASVs, respectively, to obtain species identity. Functional analysis of fungal ASV tables was performed using the FUNGuild online tool [45], an open annotation tool for parsing fungal community datasets by ecological guild.

.

## Results

### Fungal diversity and community structures in liverwort thalli were more affected by the surrounding environment when compared to prokaryotic communities

**Fig. 2:**
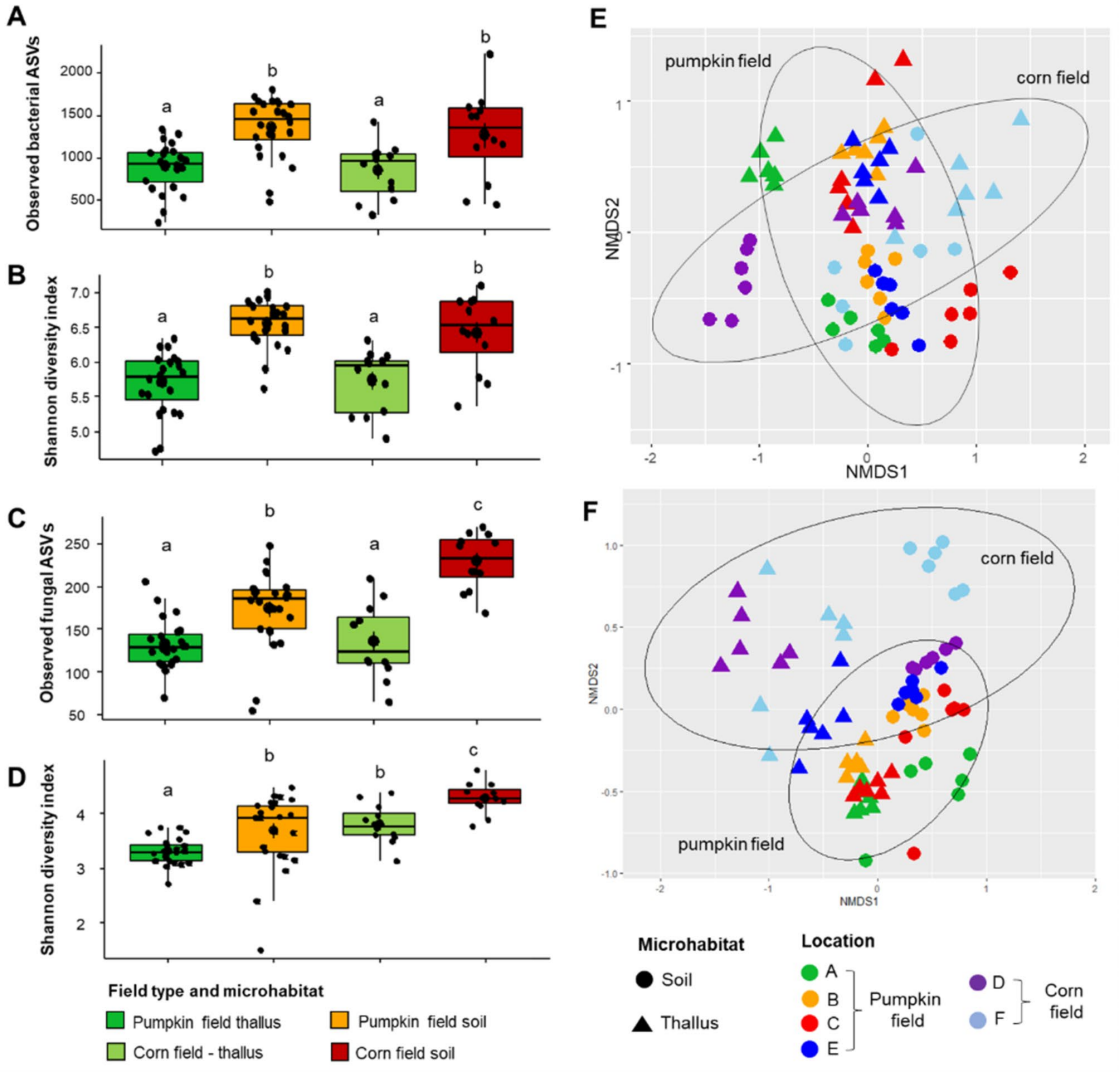
Alpha and beta diversity comparisons of thallus and liverwort samples that were obtained from corn and pumpkin fields. Prokaryote and fungal species richness (**A** - prokaryotes and **C** - fungi) and diversity according to Shannon index (**B** - prokaryotes and **D** - fungi). Bray–Curtis distance matrices of prokaryotic (**E**) and fungal (**F**) community structures between samples were visualized using nMDS plots

The microhabitat (surrounding soil vs. thallus) was found to influence microbial richness and diversity. A higher species richness and diversity of prokaryotes were observed in adhering soil samples (observed ASVs: 1327; Shannon diversity index: 6.51) in comparison to thallus samples (observed ASVs: 884; Shannon diversity index: 5.72, *P* < 0.001, Fig. 2A and B). A similar pattern was also observed for fungal species richness and diversity between adhering soil (observed ASVs: 192; Shannon diversity index: 3.90) in comparison to thalli (observed ASVs: 132; Shannon diversity index: 3.47, *P* < 0.001, Fig. 2C and D). These results indicated that species richness and diversity were lower in liverwort thalli than in adhering soil. When thallus samples were compared, species richness (*P*_adjusted_=0.905) and diversity (*P*_adjusted_=0.798) of prokaryotes in liverwort thalli did not differ between samples that were obtained from pumpkin and corn fields. However, differences in fungal diversity in liverwort thalli that were obtained from different fields were observed (*P*_adjusted_=0.008). Liverwort samples that were collected from corn fields had a higher fungal diversity (Shannon diversity index: 3.80) in comparison to those that were collected from pumpkin fields (Shannon diversity index: 3.31). Interestingly, a similar pattern also was observed when comparing fungal diversity in soil samples that were collected from the two fields (corn fields - Shannon diversity index: 4.29; pumpkin fields - Shannon diversity index: 4.29). These results suggested that the fungal diversity was more affected by the surrounding environment compared to the prokaryotic diversity.

The Adonis analysis indicated that the two tested factors, i.e., microhabitat and field type and their interactions, had a significant effect (*P* < 0.001) on the prokaryotic and fungal diversity (4.1-15.8%, *P* < 0.05; Table 1). However, only a small percentage (4.1%) of prokaryotic community variation was explained by field type whereas a higher percentage (14.9%) of prokaryotic community variation was explained by microhabitat. Supporting the Adonis analysis, a clear clustering of samples that originated from the same field type was not apparent (Table 1; Fig. 2E). A nMDS plot indicated a clear clustering between soil and thallus samples (Supplementary Figure S2A). For instance, thallus samples that were collected from location D (harvested corn fields) showed a tendency to cluster together with other thallus samples that were collected from locations B and E (harvested pumpkin fields). The microhabitat significantly influenced the fungal community structure and explained a moderate percentage (12.8%) of fungal community variation. A nMDS plot indicated a clear clustering between soil and thallus samples (Supplementary Figure S2B). Interestingly, the field type significantly affected the fungal community structure (*P* < 0.001) and explained 34.1% of fungal community variation. The nMDS plot revealed a separated cluster between soil and thallus samples that were collected from corn and pumpkin fields was also apparent (Fig. 2F). For instance, thallus samples that were collected from pumpkin fields, especially samples from locations A, B and C, clustered together. Here, Mantel tests revealed that the Bray Curtis distances between fungal communities in liverwort thalli are highly correlated with those in surrounding soil (r= 0.476, *P* <0.001). In contrast, Bray Curtis distances of prokaryotic communities in liverwort thalli only showed a weak correlation with those in surrounding soil (r= 0.182, *P* = 0.021). These results indicate that surrounding soil was the major driver of fungal community assembly in liverwort thalli. A significant correlation was also observed between the bacterial community and fungal community compositions in thalli (*P* < 0.001, *r =* 0.411). Additionally, hierarchical clustering of the fungal communities showed a higher variation between thallus samples that were obtained from different field types (Supplementary Figure S3B). Unlike the fungal communities, hierarchical clustering of the bacterial communities revealed two major clusters consisting of thallus and soil samples regardless of the field type (Supplementary Figure S3A). In the deepening analysis of liverwort thalli samples, field types explained 30.1% of fungal community variation but only 13.8% of bacterial community variation. The results indicate that bacterial communities in liverwort thalli from different field types are more stable in relation to fungal communities, which were highly affected by the field type.

**Table 1:** **Effects of microhabitat, field type, and their interaction on prokaryotic and fungal community structure (β-diversity)**

Factor	Microbial community similarities			
	Prokaryotic community		Fungal community	
	R^2^ value	*P* value	R^2^ value	*P* value
Microhabitat (M)	0.149	0.001*	0.128	0.001*
Field type (T)	0.041	0.001*	0.341	0.001*
M * T	0.074	0.001*	0.115	0.001*

*Significant differences (*P* ≤ 0.05) were assessed with the Adonis test

### Field type affected methylobacterial abundance but did not influence fungal and archaeal abundance in liverwort thalli

**Fig. 3:**
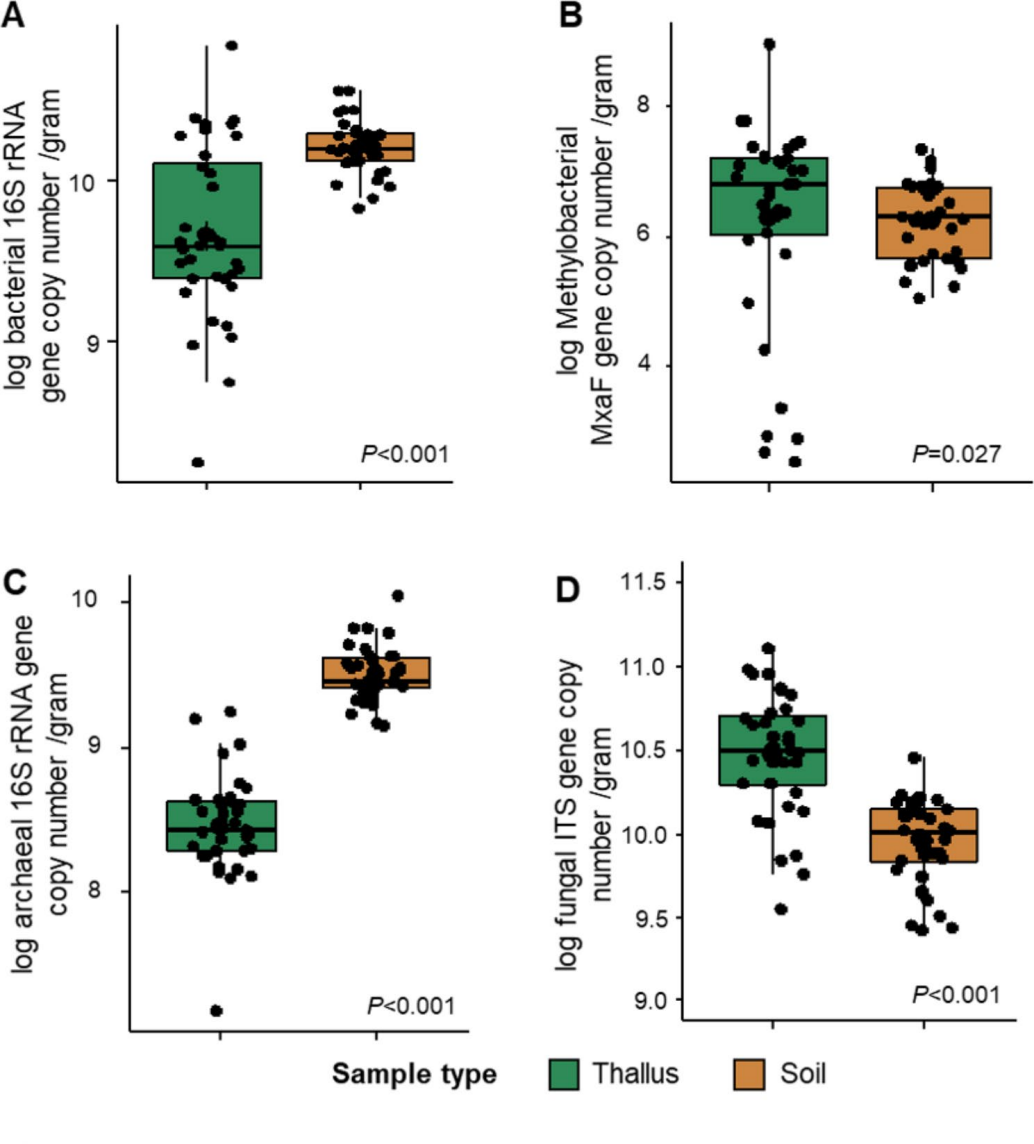
Comparisons of microbial abundances between soil and liverwort thalli. Bacterial (**A**), methylobacterial (**B**), archaeal (**C**), and fungal (**D**) abundances were quantified using a qPCR-based approach and transformed into log values. Significances in microbial abundances between soil and liverwort thalli were determined by the Kruskal Wallis test (*P* < 0.05) was used to test. Data are presented as the mean for each group

Soil samples generally contained a higher bacterial (*P* < 0.001) and archaeal (*P* < 0.001) abundance in comparison to liverwort thalli (Fig. 3A C). In contrast, a higher methylobacterial and fungal abundance was observed in liverwort thalli (*P* < 0.001, Fig. 3B and D). Abundances of bacteria, methylobacteria, archaea and fungi in soil were relatively similar between different field types (harvested pumpkin field vs. harvested cornfield, Supplementary Fig. 3). The field type significantly affected bacterial abundance in liverwort thalli (*P* < 0.001). A higher bacterial abundance was observed in those samples that were collected from harvested pumpkin fields (copy number of genes/gram: 1.26 × 10^10^, Supplementary Figure S4A) in comparison to those that were obtained from harvested corn fields (copy number of genes/gram: 4.08 × 10^9^).

A similar pattern was also observed regarding the abundance of methylobacteria in liverwort thalli between the different field types (Supplementary Figure S4B). When location-specific samples were analyzed separately, samples that were collected from locations A, B and C (1.8 × 10^7^, 1.9 × 10^7^, and 1.7 × 10^8^ copy number of MxaF genes/gram, respectively), which were harvested pumpkin fields, generally contained a higher abundance of methylobacteria in comparison to locations D and F (1.1 × 10^7^ and 1.5 × 10^5^ copy number of MxaF genes/gram, respectively) which were harvested corn fields. The ratio between methylobacteria and bacteria (ratio methylobacterial MxaF gene copy number and bacterial 16S rRNA gene copy number) was higher in thallus samples (Supplementary Figure S5A). Interestingly, the ratio was also higher in thallus samples that were collected from pumpkin fields in comparison to thallus samples that were collected from corn fields. Therefore, the increase of bacterial abundance in liverwort thalli could be mainly related to an increase of methylobacterial abundance. These results indicated that liverworts enriched methylobacteria while their abundances were also driven by the field type. There were no differences in archaeal (*P* = 0.821) and fungal (*P* = 0.718) abundances in liverwort thalli between samples that were collected from harvested pumpkin and corn fields, respectively.

### A core microbiome was identified in the liverwort thalli across field type and location

Due to a higher similarity of bacterial community structure in comparison to fungal community structures in liverwort thallus that collected from different fields, we expected a higher number of bacterial ASVs which are defined as a core liverwort thalli microbiome in comparison to fungal ASVs. The core bacterial community of liverwort thalli was composed of 38 bacterial ASVs (Supplementary Figure S6A); most members of the core bacterial community belonged to *Alphaproteobacteria* (n = 14 ASVs) i.e., *Sphingomonas* and *Rhizobium* and *Gammaproteobacteria* (n = 10 ASVs) i.e., *Rhizobacter* and *Massilia*. They contributed to an average of 19.9% of the total reads. Interestingly, five ASVs that were defined as members of the core bacterial community and were assigned to *Oxyphotobacteria* contributed to 9.4% of the total reads in thallus samples. Only 13 fungal ASVs were identified as members of the core fungal community (Supplementary Figure S6B); they were mostly assigned to *Dothideomycetes* (n = 4 ASVs) and *Tremellomycetes* (n = 4 ASVs).

**Fig. 4:**
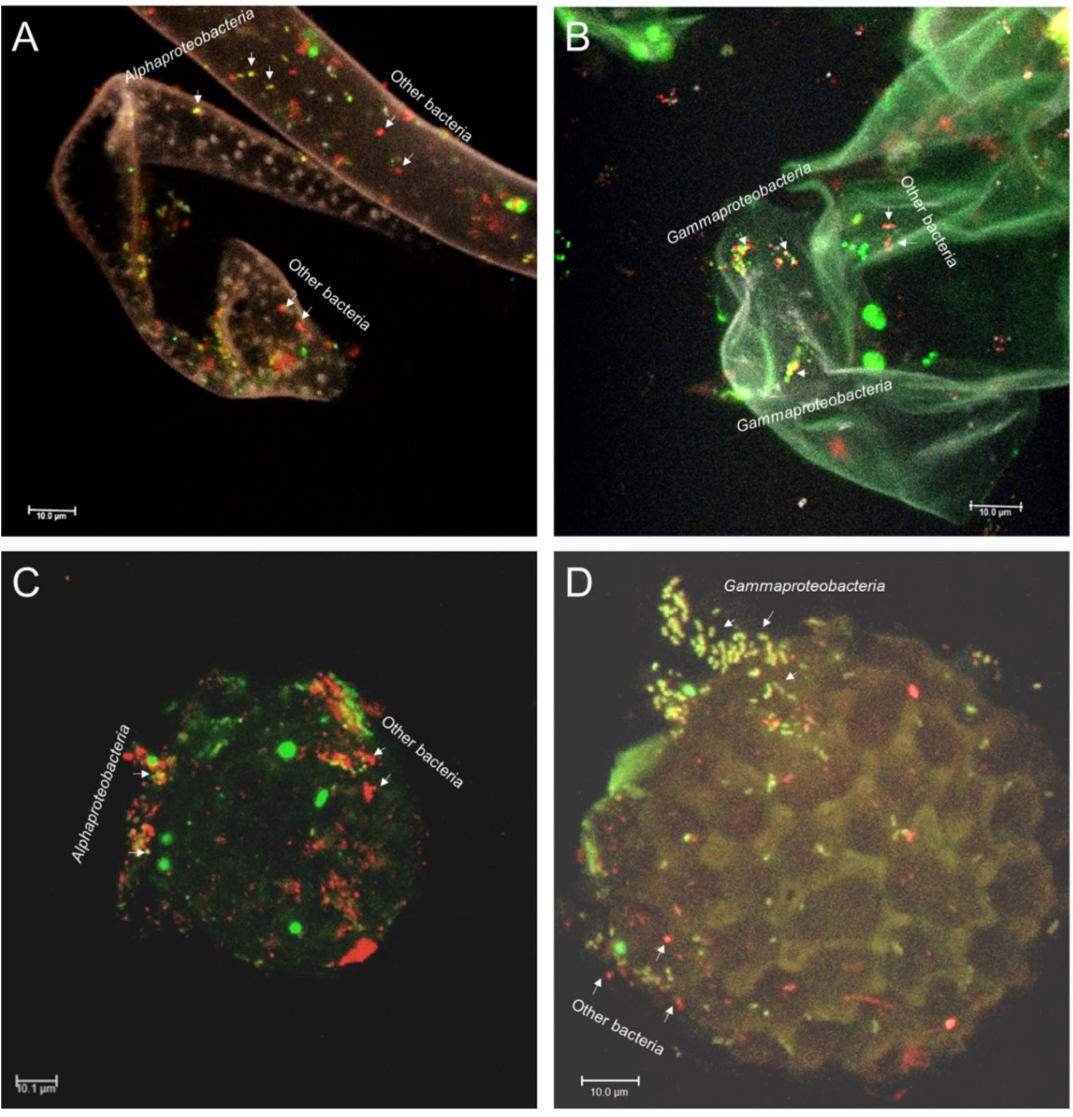
Micrographs obtained with confocal laser scanning microscopy (CLSM) in combination with fluorescence in situ hybridization (FISH) showing bacterial colonization of liverwort thalli (**A** and **B**) and spores (**C** and **D**). Bacteria were stained with FISH probes that are specific for *Alphaproteobacteria* (**A** and **C**, yellow dots), *Gammaproteobacteria* (**B** and **D**, yellow dots) and a general probe for other bacteria (**A**-**D**, red dots)

Due to the identified stability of bacterial communities in liverwort samples and indications that they were less affected by the surrounding environment, it can be assumed that they are at least partially vertically transmitted. To provide further reinforce this hypothesis, confocal laser scanning microscopy (CLSM) in combination with fluorescence in situ hybridization (FISH) was employed to visualize labeled bacteria in liverwort thalli and spores. As a washing step was included to remove soil-derived bacteria from the thallus surface, the observed bacterial cells were likely associated to *Riccia*. Bacterial communities were shown to colonize the thallus surface in a biofilm-like manner (Fig. 4). The predominance of *Alphaproteobacteria* and *Gammaproteobacteria* on liverwort thalli was confirmed with the implemented FISH-CLSM experiments (Fig. 4A and B). By using taxon-specific probes for *Alphaproteobacteria* and *Gammaproteobacteria* in combination with universal probes for other bacteria, the two specifically labeled taxa were found to be distributed in small clusters along thalli together with other bacteria. Interestingly, colonization of *Alphaproteobacteria* and *Gammaproteobacteria* was also observed on the surfaces of the liverwort spores indicating their vertical transmission (Fig. 4C and D).

### Fungal members form an accessory microbiome that is environment specific

Specific differences in bacterial composition between liverwort thalli and adhering soil were observed. At the phylum level, *Proteobacteria* was the most dominant taxon (39.3%) in both microhabitats (thallus and surrounding soil). Major differences between the two microhabitats were reflected by higher relative abundances of *Cyanobacteria* (23.0%) and *Bacteroidetes* (13.6%) in thallus samples in comparison to surrounding soil samples (5.8% and 8.8%, respectively). In contrast, a higher relative abundance of *Acidobacteria* (10.1%) and *Actinobacteria* (10.4%) was found in surrounding soil samples in comparison to thallus samples (3.8% and 3.3%, respectively). *Archaea* (phylum *Thaumarchaeota*) contributed to 3.3% and 1.4% of total reads in soils that were collected from corn and pumpkin fields, respectively. Their abundances were low in the thallus samples that were collected from the corresponding fields (0.6% and 0.3%).

**Fig. 5:**
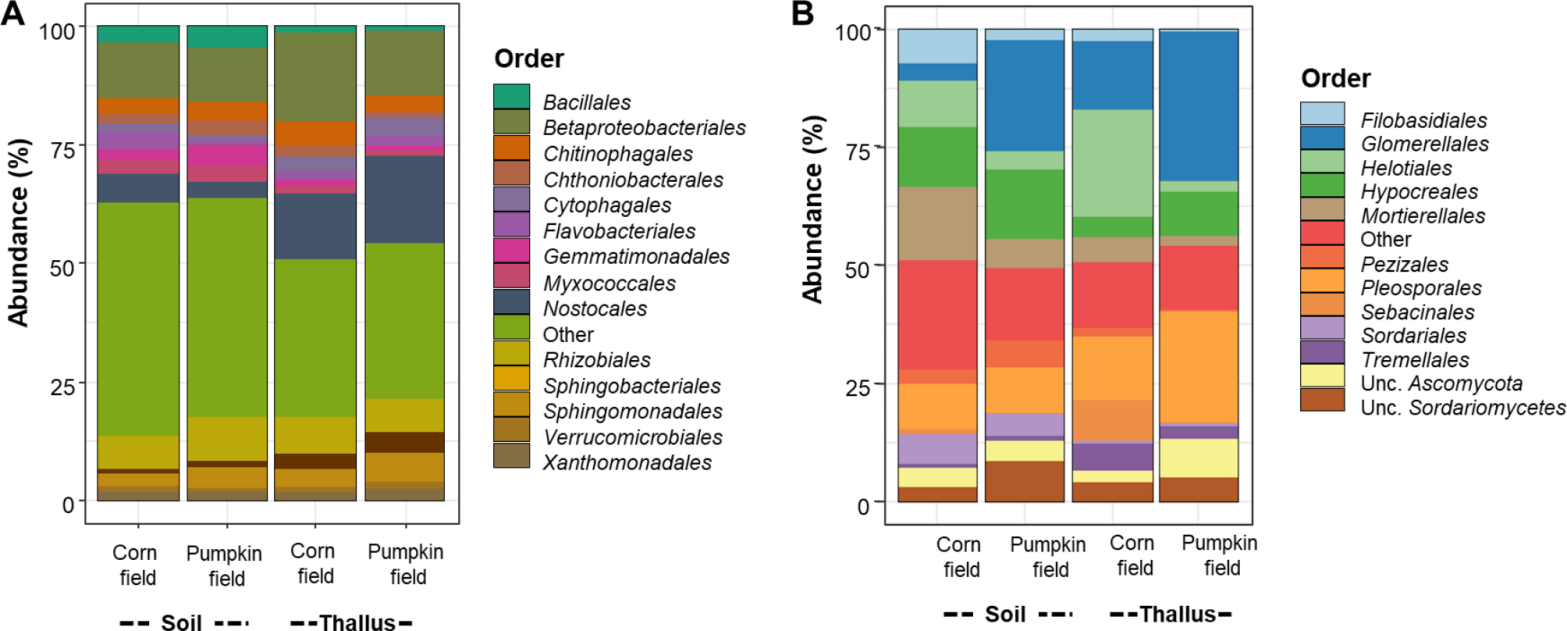
Microbial community composition. The relative abundance of distinct taxa in prokaryotic (**A**) and fungal (**B**) communities were visualized on order level

At the order level, *Nostocales* dominated the bacterial community composition in thallus samples that were collected from corn (14.0%) and pumpkin (18.4%) fields whereas this order only contributed to 6% and 3.5% of the total bacterial community in surrounding soil samples from the same field types (Fig. 5A). Furthermore, the relative abundance of *Sphingobacteriales* and *Oxyphotobacteria incertae sedis* in thallus samples was at least three-fold higher compared with that in the surrounding soil samples (Fig. 5A). We identified several bacterial taxa that had different relative abundances between the two field types. For instance, the relative abundances of *Sphingomonadales* and *Caulobacterales* were higher in thallus samples that were collected from pumpkin- (6.1% and 1.1%, respectively) and corn (3.8% and 0.7%, respectively) fields. In contrast, *Betaproteobacteriales*, *Chitinophagales* and *Chthoniobacterales* showed an opposite trend.

Differences in fungal community composition were driven by the field type. Two fungal phyla, *Ascomycota* and *Basidiomycota*, dominated soil and thallus samples by contributing to 91.2% of the total reads. Interestingly, differences at the phylum level were apparent between different field types (corn fields vs. pumpkin fields) regardless of the microhabitat. For instance, the relative abundance of *Ascomycota* was highly similar between thallus and soil samples that were collected from the same field type (pumpkin field thalli: 85.9% and pumpkin field soil: 83.5%; corn fields thallI: 64.6% and corn field soil: 69.2%). At the order level, relative abundances of the five fungal taxa *Glomerales*, *Helotiales*, *Sebacinales*, *Tremellales* and *Pleosporales* differed between thallus samples that were collected from pumpkin and corn fields (Fig. 5B). Relative abundances of *Glomerales* and *Pleosporales* were at least twofold higher in thallus samples that were collected from pumpkin fields in comparison to thallus samples that were collected from corn fields. Relative abundances of these fungal taxa were also higher in thallus samples that were collected from pumpkin fields in comparison to samples from the surrounding soil. In contrast, higher relative abundances of *Helotiales*, *Sebacinales*, and *Tremellales* were found in thallus samples that were collected from corn fields in comparison to thallus samples that were collected from pumpkin fields.

In addition, SourceTracker analysis was used to identify if the surrounding environment is a potential reservoir for the detected microbial communities in liverwort thalli. Fungal communities of liverwort thalli contained a high proportion of members that were predicted to originate from soil (mean value 71.2%, Supplementary Figure S7B), except for samples that were collected from location D. A relatively lower proportion of bacterial community members were predicted to have originated from soil and are trackable to the liverwort thalli (mean value 30.4%, Supplementary Figure S7A). These results suggest that the assembly of fungal communities in liverwort thalli was driven by the surrounding environment in comparison to the assembly of prokaryotic communities.

### *Riccia* liverworts are a reservoir for plant beneficial and pathogenic bacteria

Since variations at higher taxonomic levels may not provide sufficient information to infer ecological importance, indicator taxa for thalli that were collected from different field types were identified at ASV level using LEfSe analysis. In total, 510 ASVs were enriched in thallus samples in comparison to the surrounding soil. The majority of these ASVs belonged to *Alphaproteobacteria* (n = 115 ASVs), *Gammaproteobacteria* (n = 99 ASVs), *Bacteroidia* (n = 97 ASVs) and *Oxyphotobacteria* (n = 76 ASVs). Among these enriched ASVs, several known beneficial bacteria genera were detected such as *Rhizobium*, *Bradyrhizobium*, *Flavobacterium*, *Sphingomonas*, *Herbaspirillus* and *Methylobacterium* (Supplementary Table S2). Interestingly, there were three enriched ASVs that are closely related to known plant pathogenic bacteria i.e., *Pseudomonas viridiflava*, *Ralstonia syzygii* and *Xanthomonas campestris* (Supplementary Table S2). There were 79 fungal ASVs that were enriched in the thallus samples in comparison to the surrounding soil samples. The majority of them belonged to *Agaricomycetes* (n = 13 ASVs), *Leotiomycetes* (n = 13 ASVs), *Sordariomycetes* (n = 12 ASVs), *Dothideomycetes* (n = 11 ASVs) and *Tremellomycetes* (n = 10 ASVs). In addition, their functional features based on different trophic modes were predicted using the FUNGuild online tool. Several fungal genera that were enriched in thallus samples in comparison to the surrounding soil samples were identified as *Alternaria*, *Psathyrella* and *Plectosphaerella* (Supplementary Table S3); they are all known as phytopathogenic fungi. Interestingly, thallus samples that were collected from corn fields enriched specific fungi such as *Peziza*, *Fusarium* and *Itersonilia* whereas thallus samples that were collected from pumpkin fields enriched other fungal genera such as *Stagonosporopsis*. On the other hand, 7 enriched fungal ASVs were identified as *Mortierella* that is known as a saprotroph.

Several putatively beneficial but also plant pathogenic members of the microbiota were identified using a cultivation dependent approach. Bacterial strains that were isolated from *Riccia* thalli using King’s B (KB) medium were assigned to two genera, namely *Pseudomonas* and *Xanthomonas* (Supplementary Table S4). Putatively beneficial bacteria were identified as *P*. *laurentiana*, *P. putida* and *P. psychrotolerans*. One isolate was found to be closely related to *P. asplenii* which is the causal agent of bacterial soft rot disease in cyclamen plants [46]. Moreover, multiple isolates (n = 13) were identified as *Xanthomonas* *translucens* which is the causal agent of bacterial leaf streak in wheat and cereal crops [47]. Overall, the combined results suggest that liverwort thalli can enrich putatively beneficial but also plant pathogenic members of the microbiota and that this can be influenced by the previously cultivated crop.

## Discussion

Fundamental ecological processes require a detailed understanding of the mechanisms involved in plant microbiome assembly and host-microbe–environment interactions. This study provides an in-depth assessment of the prokaryotic and fungal diversity in liverworts, which are an integral part of natural environments. The findings indicate that methylobacteria, *Cyanobacteria* and *Bacteroidetes* and a large proportion of the fungal community are acquired from the surrounding environment. While the fungal community is influenced by the environment, bacterial communities are conserved. In addition to the well-known plant beneficial character of the majority of the bacterial inhabitants, this result indicates a symbiotic interaction of *Riccia* species with bacteria instead of mycorrhiza. Interestingly, *Riccia* liverworts may not only serve as a reservoir for plant beneficial microorganisms but also for plant pathogens. Overall, this study provides evidence for a specific and stable bacterial community in liverwort thalli while fungi likely form an accessory microbiome that is environment specific.

Taxon-specific quantifications via qPCR indicated an enrichment of methylobacteria in liverwort thalli in comparison to the surrounding soil. It was also observed that the absolute abundance of methylobacteria in surrounding soil samples from different field types was relatively similar. Hence, the enrichments of methylobacteria are likely driven by the host organism depending on the surrounding environment. This result is also supported by the higher relative abundance of the genus *Methylobacterium* that was found in liverwort thalli based on amplicon sequencing (Supplementary Figure S5B) despite its low abundance in soil and liverwort thalli. Several foregoing studies have shown positive effects of methylobacteria on bryophytes as well as specifically on liverworts [48, 49]. Various members of this bacterial group can exchange specific metabolic products e.g., growth hormones and ammonium with their host plant, leading to enhanced growth [50]. Moreover, a growing body of knowledge highlights the importance of low-abundant taxa for ecosystem functioning, i.e., enzymatic activity in soils, protection against various disturbances, and involvement in biogeochemical cycles [51–54]. Therefore, the observed enrichment of methylobacteria likely plays a role in terms of supporting host plant fitness.

Interestingly, the enrichment of methylobacteria was also driven by the field type. A previous study reported that land use affected the abundance of such bacteria on leaf surfaces [55]. In this study, one plausible explanation for the variation in the absolute abundance of methylobacteria between different field types could be due to differences in nutrient availability. It is worth to note that methylobacteria are highly adapted to survive in low-nutrient environments (i.e., oligotrophic) due to their genetic repertoire that enables the production of carotenoids, reactive oxygen species and extracellular polysaccharides [56, 57]. Accumulation of nutrients including mineral elements in the thalli from surrounding soil could affect metabolic activity in liverworts e.g., methanol and methylamine production [12, 14] which then could determine the abundance of methylobacteria.

The present study also provides evidence for a high specificity of bacterial communities associated with liverworts and indicates that surrounding soil is not a major resource for them. Previously, specific as well as functionally adapted bacterial communities were found in different bryophytes including liverworts [9]. Bryophytes are known to maintain a specific bacterial community within the whole lifecycle, independent of their geographic location [58, 59]. The maintenance of such bacterial communities can be partially explained by vertical transmission via spores; this is in analogy to trans-generational transfer of microbes in seeds of vascular plants [60–62]. Deepening analyses conducted in the frame of this study indicated that all samples shared a core microbiome consisting of *Oxyphotobacteria* (phylum *Cyanobacteria*), *Alphaproteobacteria and Gammaproteobacteria.* This subset of bacterial ASVs with a relatively high abundance (an average of 19.9% of the total reads) was consistently detected in liverwort thalli independent of the geographical location. Core bacterial community members with high relative abundance might play essential roles for their host’s physiology [63]. *Cyanobacteria*, which were present in the core microbiome, were previously suggested as an important group of bryophyte symbionts due to their potential to provide nitrogen to their host [64].

The fungal diversity and community structure in liverwort thalli were mainly affected by the surrounding environment indicating that the identified fungi may form an accessory microbiome that is environment specific. In contrast to the more stable prokaryotic community, a strong influence of the field type on fungal diversity and the community structure was observed. Previous studies have already demonstrated the effect of cultivation history on endophyte and rhizosphere microbial diversity [65–67]. For instance, in this study, we observed a high abundance of *Glomerales*. Previous studies showed that mycorrhizal colonization in sweet corn (*Zea mays*) and pumpkin (*Cucurbita pepo*) can range from moderate to high [68–71]. It is possible that the crops cultivated on the analyzed fields have left an imprint on the local soil microbiome, which is then transferred to liverwort thalli. Taken altogether, crop cultivation history could indirectly drive selection of fungal communities in liverwort thalli via surrounding soil reservoirs.

Interestingly, ASVs that were assigned to known bacterial and fungal phytopathogens, were enriched in the thallus samples in comparison to the surrounding soil. These ASVs included *Pseudomonas viridaflava*, *Xanthomonas* *translucens*, *Xanthomonas campestris* pv. campestris and *Alternaria brassicae*. All these taxa are known to have the ability to survive in the environment outside of their host plants and infect a broad range of plant species [47, 72–74]. Moreover, *Altenaria* and *Stagonosporopsis* are known to cause disease in pumpkin and maize; these crops were previously grown at the sampling sites [75, 76] and may have thus facilitated an enrichment of the pathogens in the liverwort thalli. Only little knowledge on pathogenic microbes that cause diseases in liverworts is available [77]. The presence of pathogenic microorganisms within the native microbiota of some plants was previously described [78, 79]. The presence of such microorganisms is normally not connected to disease emergence in non-host plants. We therefore suggest that liverworts may play a so far undescribed role as reservoirs of bacterial and fungal pathogens that may be transmitted to crop plants grown at the same location. Despite the fact that specific ASVs and sequences of isolated strains were closely related to sequences of known pathogens, we acknowledge that this approach is not sufficient to provide evidence for their pathogenicity or beneficial properties. Confirming such implications would require targeted microbial inoculation experiments with plants. In addition, experiments such as multi-series sampling to investigate population dynamics of the pathogen in *Riccia* using qPCR and pathogenicity assays will be useful to further elucidate such implications. We also acknowledge that more efforts are required, i.e., implementation of different media and incubation conditions, to maximize the recovery of culturable bacteria, especially of those that are less abundant and recalcitrant. This will enable us to obtain a holistic picture of the diversity and functions of cultivable Riccia-associated bacteria.

In conclusion, this study revealed the presence of a stable bacterial community but varying fungal communities in liverwort thalli. Furthermore, the enrichment of fungal taxa was driven by the field type that is likely due to an imprint on soil microbiomes by the cultivated crop plants. This study also provides a basis for deepening analyses targeting functional implications of the *Riccia* microbiome, which is currently underexplored, and serve as a potential model to answer plant microbiome assemblages of evolutionary old land plants.

## Electronic supplementary material

Below is the link to the electronic supplementary material.

Supplementary Material 1

## Data Availability

The amplicon sequencing dataset has been deposited in the European Nucleotide Archive (ENA) database under the study number PRJEB52587.
