# Supplementary material for: The microbiome of Riccia liverworts is an important reservoir for microbial diversity in temporary agricultural crusts
Source: Environ Microbiome. 2023 Jun 1;18:46. doi: 10.1186/s40793-023-00501-0 (PMC10233997; doi:10.1186/s40793-023-00501-0)
Supplement: Supplementary file 1 — Supplementary Material 1 [file 40793_2023_501_MOESM1_ESM.docx]

**Supplementary Material**

**The microbiome of *Riccia* liverworts is an important reservoir for microbial diversity in temporary agricultural crusts**

**Wisnu Adi Wicaksono^1^, Bettina Semler^1^, Martina Pöltl^2^**, **Christian Berg^2^, Gabriele Berg^1,3,4^, Tomislav Cernava^1#^,**

^1^ Institute of Environmental Biotechnology, Graz University of Technology, 8010 Graz, Austria

^2^ University of Graz, Institute of Biology, 8010 Graz, Austria

^3^ Leibniz Institute for Agricultural Engineering and Bioeconomy (ATB), Potsdam, Germany

^4^ Institute for Biochemistry and Biology, University of Potsdam, Potsdam, Germany

^#^Corresponding author:

Tomislav Cernava, Graz University of Technology, Graz

Emails: tomislav.cernava@tugraz.at

**Authors’ email addresses**

[wisnu.wicaksono@tugraz.at](mailto:wisnu.wicaksono@tugraz.at), bettina.semler@gmail.com, martina.poeltl@museum-joanneum.at, christian.berg@uni-graz.at, gabriele.berg@tugraz.at

**Short title:** Liverworts microbiome

**Submitted to: Environmental Microbiome**

**Supplementary Table S1. Details of sampling locations**

| Sample ID | Species | Location | Field type |
| --- | --- | --- | --- |
| A 1-6 | *Riccia bifurca* | Steiermark, Hausmannstätten, 1,0 km NW Enzelsdorf | pumpkin field |
| B 1-6 | *Riccia bifurca* | Steiermark, Hartberg, 2 km NNE Wörth an der Lafnitz | pumpkin field |
| C 1-6 | *Riccia bifurca* | Steiermark, Preding, 690 m SE Mettersdorf | pumpkin field |
| D 1-6 | *Riccia glauca* | Steiermark, Hausmannstätten, 1,1 km NNE Fernitz | corn field |
| E 1-6 | *Riccia bifurca* | Burgenland, Güssing, 1.5 km SW Wörtherberg | pumpkin field |
| F 1-6 | *Riccia bifurca* | Steiermark; Eggersdorf bei Graz, 1.1 km W Brodersdorf | corn field |

**Supplementary Table S2.** List of bacterial ASVs that were significantly enriched in thallus samples in comparison to the surrounding soil according to linear discriminant analysis effect size (LEfSe) analysis

| ASV ID | LDA score | Taxonomical information | | Relative abundance (%) | |
| --- | --- | --- | --- | --- | --- |
|  |  | Class | Genus | |  |
| 0f74a957d2a112908a68b9ad19d1da6d | 3.8 | Nitrososphaeria | Unc. Nitrososphaeraceae | | 0.024 |
| 85fca7d0ba5e9b3a3198961bd9557f18 | 3.9 | Nitrososphaeria | Unc. Nitrososphaeraceae | | 0.028 |
| c4907d9038f53a0d8a39b0c156c5b9bc | 3.8 | Blastocatellia_(Subgroup_4) | Unc. 11_24 | | 0.023 |
| f7f3f2e319eb0bbe833cec1454ce0787 | 3.9 | Blastocatellia_(Subgroup_4) | Aridibacter | | 0.053 |
| a652f496fb4f9105e6797d717c3926ef | 3.7 | Blastocatellia_(Subgroup_4) | Unc. Blastocatellaceae | | 0.100 |
| 135c54fd0b2c2cd074f7be044efce948 | 3.6 | Subgroup_6 | Unc. Subgroup_6 | | 0.023 |
| 67588f6f0b9b141bc5f37204a50da5bf | 4.0 | Subgroup_6 | Unc. Subgroup_6 | | 0.049 |
| 69304d479a6cbf8e4d348d51bbdffe7a | 3.3 | Subgroup_6 | Unc. Subgroup_6 | | 0.030 |
| 956f15a69b1fac98cc58d6143a5d75d8 | 3.8 | Subgroup_6 | Unc. Subgroup_6 | | 0.022 |
| b1a795217b9a3f4814ee5cae27450501 | 3.5 | Thermoanaerobaculia | Subgroup_10 | | 0.103 |
| 09de3dde6c465b74bcb8e6f8a478e4e0 | 3.2 | Actinobacteria | Rhodococcus | | 0.040 |
| e8ccf9dcc883d1d3cef7bb69235bfdda | 3.4 | Actinobacteria | Actinoplanes | | 0.038 |
| b3b99c7e3a0d655fd4aeadf93820978c | 3.1 | Actinobacteria | Nocardioides | | 0.029 |
| e7f82f35bba1e81dafa173bf71a879cf | 3.1 | Armatimonadia | Unc. Armatimonadales | | 0.056 |
| 4258f737dc7eddabe5676f2de2276614 | 3.4 | Fimbriimonadia | Unc. Fimbriimonadaceae | | 0.124 |
| 4d7dd0cbb43d17d729f234f2f0bf62cd | 3.9 | Bacteroidia | Flavihumibacter | | 0.033 |
| af4f82920f7e7ab5aaea052daf84e26b | 4.1 | Bacteroidia | Lacibacter | | 0.042 |
| a0b9c413db1dc97e273db823b71d0a07 | 3.5 | Bacteroidia | Sediminibacterium | | 0.040 |
| 8f71a7b9dc989c5b4688583a2111651f | 3.9 | Bacteroidia | Terrimonas | | 0.052 |
| 2eaaa4a12d530b11bcd95a69a80646e2 | 3.2 | Bacteroidia | Unc. Chitinophagaceae | | 0.039 |
| 33b680eee4f8b01a15a33f7fe5e0596c | 3.0 | Bacteroidia | Unc. Chitinophagaceae | | 0.045 |
| 5dd9b4c4fb4de32a93a6e834dde0faa2 | 3.2 | Bacteroidia | Unc. Chitinophagaceae | | 0.026 |
| 64bd319e4d15d6e8c2d76f097883303c | 3.8 | Bacteroidia | Unc. Chitinophagaceae | | 0.037 |
| 8678dea077ece20287b950cd5bc66db8 | 3.8 | Bacteroidia | Unc. Chitinophagaceae | | 0.047 |
| 8e66bb13463efd8fe5ef1bfadc38d0a6 | 3.5 | Bacteroidia | Unc. Chitinophagaceae | | 0.030 |
| edb114398ced5bf958f4f404493a6642 | 3.9 | Bacteroidia | Haliscomenobacter | | 0.103 |
| d1fab8bc86502174bd144adb087a852e | 3.2 | Bacteroidia | Unc. Saprospiraceae | | 0.039 |
| 44245871134a7a84ac8e3049b9769980 | 3.7 | Bacteroidia | Rhodocytophaga | | 0.048 |
| 3e39c317a82e348f46ebe8943a3df168 | 3.2 | Bacteroidia | Hymenobacter | | 0.023 |
| 5f7326bcb7c2a6349f99a07e09085430 | 3.9 | Bacteroidia | Flexibacter | | 0.056 |
| a8a8751d72c8d3e4fe1bf8f149027d79 | 4.2 | Bacteroidia | Flexibacter | | 0.121 |
| 4cc867835a4167f163fa7da41d843281 | 3.5 | Bacteroidia | Unc. Microscillaceae | | 0.028 |
| 60c5e21779c0b5b385d77df36cdcf45b | 3.4 | Bacteroidia | Unc. Microscillaceae | | 0.146 |
| b6aa0321169856fa2d98ea9a21520b5a | 3.6 | Bacteroidia | Unc. Microscillaceae | | 0.065 |
| ba9ecb28932826e70d43988826679fbc | 3.4 | Bacteroidia | Unc. Microscillaceae | | 0.020 |
| 8ba8d462bcfca4f3dffd540c2595af0e | 4.3 | Bacteroidia | Arcicella | | 0.164 |
| 1ebf3c81b9f872f5a309a1a08ab97d1a | 3.4 | Bacteroidia | Dyadobacter | | 0.107 |
| b69cb20337bec667ab64a7cf2c5cea83 | 4.1 | Bacteroidia | Dyadobacter | | 0.039 |
| eebca187d104e314ca21d1c61cd5ad31 | 4.1 | Bacteroidia | Dyadobacter | | 0.055 |
| cb7242fb2a2ccdda6e2ead2c5f13e097 | 3.5 | Bacteroidia | Lacihabitans | | 0.085 |
| 025f8d52bf0fe507d3355b96f306b99b | 3.9 | Bacteroidia | Runella | | 0.096 |
| 090540da987889f054f49b2eea04ffd8 | 2.8 | Bacteroidia | Spirosoma | | 0.049 |
| 5294c0e2c5ab571842b07c413291a71e | 3.3 | Bacteroidia | Spirosoma | | 0.057 |
| 4db5730d0bbca58a8ecb6d9d5352f5bb | 3.5 | Bacteroidia | Flavobacterium | | 0.049 |
| 69dc82f398c9aa68a819235b4bf0ffd4 | 4.0 | Bacteroidia | Flavobacterium | | 0.041 |
| 85a3d7550bf533f1cdbb64dfb47d699a | 4.0 | Bacteroidia | Flavobacterium | | 0.031 |
| a26da340960942d894b28559f2430838 | 3.9 | Bacteroidia | Flavobacterium | | 0.053 |
| fca1ed7792b0c80cd00c60203ba400e8 | 3.7 | Bacteroidia | Flavobacterium | | 0.041 |
| 12dd6509c32da6a9937d21f5b52f07dc | 3.7 | Bacteroidia | Unc. env.OPS_17 | | 0.087 |
| 4313306b43246178431cfd48a6a6850a | 3.1 | Bacteroidia | Mucilaginibacter | | 0.203 |
| a531dc57776d2c54e4dd31f15d2e3517 | 3.7 | Bacteroidia | Mucilaginibacter | | 0.026 |
| ec2fa165297294eadaa031c1ded90990 | 4.5 | Bacteroidia | Mucilaginibacter | | 0.277 |
| efd7180718b7d32861503979452ea9ee | 3.6 | Bacteroidia | Mucilaginibacter | | 0.055 |
| 0d34e514b2d0d3a14fbfa27a9717f5ae | 3.3 | Bacteroidia | Pedobacter | | 0.028 |
| 21727479c0ea68be39ae81351790bc55 | 3.6 | Bacteroidia | Pedobacter | | 0.038 |
| 861c357388b498ed9ef7ef6440b4dffa | 3.0 | Bacteroidia | Pedobacter | | 0.030 |
| eed4445c6828c17a10169e46afa6fa5c | 3.6 | Bacteroidia | Pedobacter | | 0.113 |
| 3ba1982f89303f12066c5e9355ea6a16 | 4.2 | Anaerolineae | Unc. A4b | | 0.050 |
| e4d0937b69713698efbb066f6eb82bce | 3.3 | Anaerolineae | Unc. A4b | | 0.022 |
| fd9f7f75931346a713bfd1fab9bbe103 | 3.4 | Anaerolineae | Unc. A4b | | 0.023 |
| 68c1b0dd626da94d2775582a1c56c3ae | 3.8 | Chloroflexia | Unc. Chloroflexaceae | | 0.114 |
| 7ba80206a0bd1b67828dcf580d7f7cae | 4.3 | Chloroflexia | Unc. Chloroflexaceae | | 0.066 |
| 00152f4ee92b88f80848c1a406e0c75f | 3.8 | Chloroflexia | Unc. JG30-KF-CM45 | | 0.022 |
| b5a75ee30645cb947ad835d87d6cfa11 | 3.2 | Chloroflexia | Unc. JG30-KF-CM45 | | 0.027 |
| e05c79009fd7877b24e3127bd49986a0 | 3.3 | Chloroflexia | Unc. JG30-KF-CM45 | | 0.040 |
| 4240cbdefb886ef8e0b16e50862861b4 | 3.6 | TK10 | Unc. TK10 | | 0.057 |
| 60c56a4328049f5adcfe005b820f4e0e | 3.7 | Oxyphotobacteria | Unc. Leptolyngbyaceae | | 0.123 |
| 8e1a6a5890d8fa172a8e163e59354352 | 3.9 | Oxyphotobacteria | Unc. Leptolyngbyaceae | | 0.029 |
| b895b57881451be1a15a9ad8ae19c77d | 4.5 | Oxyphotobacteria | Unc. Leptolyngbyaceae | | 0.099 |
| ac224dcace889a6d0aba2c7370cea1e2 | 3.9 | Oxyphotobacteria | Unc. Coleofasciculaceae | | 0.030 |
| eb8d2f053f7543470dbc92ccdec4a732 | 3.7 | Oxyphotobacteria | Unc. Coleofasciculaceae | | 1.266 |
| f7f8b063ce4a5a492dd81a6691416982 | 3.9 | Oxyphotobacteria | Unc. Coleofasciculaceae | | 0.027 |
| 3e4fa9b6325c9bde8be2fae806c05a06 | 3.7 | Oxyphotobacteria | Desmonostoc_PCC-7422 | | 0.036 |
| 63f3ef8d5630be1b78efd960c93b16de | 4.3 | Oxyphotobacteria | Desmonostoc_PCC-7422 | | 0.072 |
| e449efdbe35238e3bdef2b82db526189 | 4.7 | Oxyphotobacteria | Desmonostoc_PCC-7422 | | 0.192 |
| 8d52cb718d903c851f933ea50736b98f | 3.9 | Oxyphotobacteria | Nostoc_PCC-73102 | | 0.027 |
| af46164da838e8da9591dba9ab73c47b | 3.9 | Oxyphotobacteria | Nostoc_PCC-73102 | | 0.028 |
| 0ae5c24423a66064004078d992f01e5a | 3.8 | Oxyphotobacteria | Nostoc_PCC-7524 | | 0.137 |
| ab96d1a4f844d82cf7a50d318ea1d9fe | 4.3 | Oxyphotobacteria | Nostoc_PCC-7524 | | 0.070 |
| 5a8847b3ca60344b74b9fddc331aafea | 3.9 | Oxyphotobacteria | Tolypothrix_PCC-7601 | | 0.037 |
| ac0758d667de4ffa5463a00b7366644e | 3.9 | Oxyphotobacteria | Tolypothrix_PCC-7601 | | 0.024 |
| 0a45522cefed356ff2c749a67addcc60 | 4.4 | Oxyphotobacteria | Unc. Nostocaceae | | 0.083 |
| 0ff572ebb03238089a34066ed9df61a2 | 4.1 | Oxyphotobacteria | Unc. Nostocaceae | | 1.759 |
| 79d0a0b0935bb622692f55cadfce2d71 | 4.3 | Oxyphotobacteria | Unc. Nostocaceae | | 0.086 |
| b87b2cdba1ced5742d13294be4ab09de | 4.3 | Oxyphotobacteria | Unc. Nostocaceae | | 0.248 |
| cadccdfb86d52c6082d051fe82b40d9e | 4.0 | Oxyphotobacteria | Unc. Nostocaceae | | 0.063 |
| f0f7f6402506e94b4d324194449decdf | 3.6 | Oxyphotobacteria | Unc. Nostocaceae | | 0.024 |
| ed6703658fa0dd8e2a34c26cfc4c6fcc | 4.5 | Oxyphotobacteria | Unc. Nostocales | | 0.123 |
| 0163abc62d56baf585a505dedd9efccf | 2.7 | Oxyphotobacteria | Unc. Oxyphotobacteria_Incertae_Sedis | | 0.230 |
| 48cbcfc08d9024dafffb2c485575c59e | 4.6 | Oxyphotobacteria | Unc. Oxyphotobacteria_Incertae_Sedis | | 0.289 |
| 7530a5618050119850926d8ede651558 | 4.5 | Oxyphotobacteria | Unc. Oxyphotobacteria_Incertae_Sedis | | 0.220 |
| 8fef6295cdbe8469d364b1ffd9785e90 | 4.3 | Oxyphotobacteria | Unc. Oxyphotobacteria_Incertae_Sedis | | 0.073 |
| 9309d02d468b5da179eee6df0e389eda | 4.2 | Oxyphotobacteria | Unc. Oxyphotobacteria_Incertae_Sedis | | 0.048 |
| d9bb002371fd4d69be8f846905705dfb | 3.8 | Oxyphotobacteria | Unc. Oxyphotobacteria_Incertae_Sedis | | 0.023 |
| dd25b975148e5aa51ca36fb002f7da5e | 4.1 | Oxyphotobacteria | Unc. Oxyphotobacteria_Incertae_Sedis | | 0.385 |
| e608fb0f86eeed97d9bf010c8d63bc13 | 5.0 | Oxyphotobacteria | Unc. Oxyphotobacteria_Incertae_Sedis | | 0.368 |
| e9ddf3ee95ee015b41923a3ada331c96 | 4.4 | Oxyphotobacteria | Unc. Oxyphotobacteria_Incertae_Sedis | | 0.227 |
| ea4944f4a6672022b58b763552fdb34f | 4.1 | Oxyphotobacteria | Unc. Oxyphotobacteria_Incertae_Sedis | | 0.041 |
| eb39bb09b42890d610424c2e2b5e48eb | 4.6 | Oxyphotobacteria | Unc. Oxyphotobacteria_Incertae_Sedis | | 0.146 |
| 1f59c7c7d6315b9eef6f6da2d7bc1d54 | 3.8 | Oxyphotobacteria | Nodosilinea_PCC-7104 | | 0.054 |
| 72997509405337509c1a935bdc038979 | 4.0 | Oxyphotobacteria | Nodosilinea_PCC-7104 | | 0.040 |
| cdb0711b3674e74cf792830728c088b4 | 4.2 | Oxyphotobacteria | Nodosilinea_PCC-7104 | | 0.056 |
| cf1d1f1c81bd074c0147e2b18a7509f5 | 4.1 | Oxyphotobacteria | Pseudanabaena_PCC-7429 | | 0.137 |
| 25fe23e989514e818f4bc1f680ad6da1 | 3.5 | Bacilli | Exiguobacterium | | 0.023 |
| 529f11e5ccc0f6a50128fefb3d4bb216 | 4.1 | Gemmatimonadetes | Gemmatimonas | | 0.041 |
| a03a07448de89a434e18e5c995194cf4 | 3.6 | Gemmatimonadetes | Unc. Gemmatimonadaceae | | 0.030 |
| 3b9a640a2fb04ef04b82b218fc1fb455 | 3.5 | Planctomycetacia | Fimbriiglobus | | 0.020 |
| 7930a1a2ee1c943cc57b41c8266c843c | 4.2 | Planctomycetacia | Fimbriiglobus | | 0.054 |
| cd69ca36ef48989ae692ab250a60f179 | 3.8 | Planctomycetacia | Gemmata | | 0.026 |
| 3c3861c581adf9b8a8cf01f20fdbaf2c | 3.7 | Planctomycetacia | Pir4_lineage | | 0.089 |
| c630b2c3c028752c233b408d0dd72656 | 3.4 | Planctomycetacia | Pirellula | | 0.026 |
| 1eebc9a9a74a9e6d87faf669f27fd6a4 | 3.6 | Planctomycetacia | Unc. Pirellulaceae | | 0.058 |
| 6c06afff9b6b2f236e0662547acd6671 | 3.8 | Planctomycetacia | Unc. Pirellulaceae | | 0.022 |
| 929e8450daa3418e6311c0a70f6aae0c | 3.6 | Planctomycetacia | SH-PL14 | | 0.040 |
| 1274879de84c6e9f91c004eb4037d06f | 3.9 | Alphaproteobacteria | Roseomonas | | 0.029 |
| de3901260547a323762fba9b9dec22ca | 3.1 | Alphaproteobacteria | Roseomonas | | 0.053 |
| 0fa9b44d52836d2f173f4ac532f8a013 | 3.6 | Alphaproteobacteria | Skermanella | | 0.048 |
| 22b545cd4650317c5335dc492e3bf8cf | 3.6 | Alphaproteobacteria | Brevundimonas | | 0.082 |
| 41e6c0b55654af1d5cd503359fb77d18 | 4.0 | Alphaproteobacteria | Brevundimonas | | 0.093 |
| a537d8bab85c83b0e74c73c55790324b | 3.3 | Alphaproteobacteria | Brevundimonas | | 0.049 |
| 573091d7ddd85f9c5f14a7e53d2ba0bf | 3.1 | Alphaproteobacteria | Caulobacter | | 0.044 |
| df14192046a031174a4ff560c358db28 | 3.2 | Alphaproteobacteria | Caulobacter | | 0.021 |
| 6ed92ab63df4b70c0ecb57cff4e0f6c8 | 3.1 | Alphaproteobacteria | Unc. Caulobacteraceae | | 0.031 |
| 5b9a650710e7f198e4be4a316a0ddfed | 4.0 | Alphaproteobacteria | Hyphomonas | | 0.036 |
| f073df90e41bdd0c9674d8de667f5f32 | 3.8 | Alphaproteobacteria | SWB02 | | 0.025 |
| f5fab172b06ecb22a6fa3a3e46aebf8b | 3.4 | Alphaproteobacteria | Unc. Micavibrionaceae | | 0.032 |
| 48cc8977d2f62f22558655e3a53d2300 | 3.6 | Alphaproteobacteria | Unc. Micavibrionales | | 0.026 |
| 6830008201e282eff938198a4d4acca8 | 3.3 | Alphaproteobacteria | Unc. Micavibrionales | | 0.077 |
| 0ae5531a9802e71139c959faebdde10c | 2.9 | Alphaproteobacteria | Reyranella | | 0.027 |
| 709a1dba0b0023a853d5cf6767864035 | 3.7 | Alphaproteobacteria | Unc. A0839 | | 0.043 |
| 4473ac847f597164696bc96da74ca753 | 3.8 | Alphaproteobacteria | Bosea | | 0.062 |
| 04ecfad5772d2e09a84a0f5ef460536c | 3.4 | Alphaproteobacteria | Methylobacterium | | 0.168 |
| 853db0ade7009a2872dc479f2465aba1 | 3.4 | Alphaproteobacteria | Methylobacterium | | 0.038 |
| a91108c92f693afd91f04b849e036d52 | 3.8 | Alphaproteobacteria | Methylobacterium | | 0.309 |
| ab6b7b90747341ce39f82d0a22a1d37d | 3.9 | Alphaproteobacteria | Methylobacterium | | 0.148 |
| e0f50c5adf537a0a3a63e61720b38ed5 | 3.1 | Alphaproteobacteria | Methylobacterium | | 0.020 |
| 398b683b106b191a01b80619fcae58ee | 3.9 | Alphaproteobacteria | Devosia | | 0.024 |
| b7f5af401f678ffbe9f7299cfa59e3bf | 3.4 | Alphaproteobacteria | Devosia | | 0.039 |
| 41b024826cbf8203352777d695f524d5 | 3.8 | Alphaproteobacteria | Hyphomicrobium | | 0.033 |
| 2653f9a6e90d957c951070035e6edeea | 3.6 | Alphaproteobacteria | Allorhizobium-Neorhizobium-Pararhizobium-Rhizobium | | 0.021 |
| 31dce60a2ad0740fbba0a68b81c80b75 | 4.0 | Alphaproteobacteria | Allorhizobium-Neorhizobium-Pararhizobium-Rhizobium | | 0.214 |
| 46ef48b6096947c86bd1a70949ca8c97 | 4.2 | Alphaproteobacteria | Allorhizobium-Neorhizobium-Pararhizobium-Rhizobium | | 0.285 |
| 4ba6bad752253e930876e8c688e4d9fb | 3.4 | Alphaproteobacteria | Allorhizobium-Neorhizobium-Pararhizobium-Rhizobium | | 0.024 |
| 5073eb042f1600783fe65118c8151bc3 | 4.0 | Alphaproteobacteria | Allorhizobium-Neorhizobium-Pararhizobium-Rhizobium | | 0.035 |
| 8b9ab15395d95e4eb8b0592b2a46be6d | 3.5 | Alphaproteobacteria | Allorhizobium-Neorhizobium-Pararhizobium-Rhizobium | | 0.043 |
| cd9bccba7f7c9aecd8eeac841bcb72cc | 3.6 | Alphaproteobacteria | Allorhizobium-Neorhizobium-Pararhizobium-Rhizobium | | 0.038 |
| e1c7d97fe13e9127d225d76a1feb8c78 | 4.6 | Alphaproteobacteria | Allorhizobium-Neorhizobium-Pararhizobium-Rhizobium | | 0.458 |
| fc6622c636a5210293fb2873fc4761d9 | 3.8 | Alphaproteobacteria | Allorhizobium-Neorhizobium-Pararhizobium-Rhizobium | | 0.110 |
| c3fe6ae58bb1e240a989dac6b2708532 | 3.5 | Alphaproteobacteria | Aureimonas | | 0.056 |
| dfad8c8bd0537264e789e7f08664f69b | 3.5 | Alphaproteobacteria | Unc. Rhizobiaceae | | 0.053 |
| 860b11244363460108e1a96df96599a1 | 3.8 | Alphaproteobacteria | Nordella | | 0.024 |
| 4d5b8c2c09a9e464f466f3ddc5ab1de5 | 3.8 | Alphaproteobacteria | Unc. Rhizobiales_Incertae_Sedis | | 0.024 |
| 00f22fe0aa7d9b976a6a884f493a1b75 | 3.9 | Alphaproteobacteria | Unc. Xanthobacteraceae | | 0.062 |
| ca8f1c260ac18a133685554fc1089155 | 3.9 | Alphaproteobacteria | Rhodobacter | | 0.076 |
| d2ac92ef7b0297714611e7160d79672a | 4.0 | Alphaproteobacteria | Rhodobacter | | 0.034 |
| 6c3fb343f46983254cdb83e99fa07a49 | 4.6 | Alphaproteobacteria | Unc. Rhodobacteraceae | | 0.428 |
| 6e0f9d60b3729c86b31a72bc587db1e7 | 4.1 | Alphaproteobacteria | Unc. Rhodobacteraceae | | 0.086 |
| 26f173c04574871aba8876838e2d9e61 | 3.4 | Alphaproteobacteria | Novosphingobium | | 0.041 |
| 3b50e9874fa67e04b49f5a59ecf0059a | 4.4 | Alphaproteobacteria | Porphyrobacter | | 0.310 |
| 0aa2e510fc63e5d46add3e8000cf0a7f | 4.7 | Alphaproteobacteria | Sphingobium | | 0.182 |
| 2bbb28b70c3a3757e48663f4d0003ecc | 4.0 | Alphaproteobacteria | Sphingobium | | 0.034 |
| 8359cc1215c0ae7475efe27dbd6cdc26 | 4.0 | Alphaproteobacteria | Sphingobium | | 0.109 |
| 0a165844daf38d8ce2008c94a5b4565e | 3.2 | Alphaproteobacteria | Sphingomonas | | 0.119 |
| 2727f819185a7e8993f13df355a399fa | 4.2 | Alphaproteobacteria | Sphingomonas | | 0.434 |
| 5d0c6af420698f1cdff612c8f7960627 | 3.4 | Alphaproteobacteria | Sphingomonas | | 0.060 |
| 8bd7e79466b5a176fbb500254a2f9c9a | 4.4 | Alphaproteobacteria | Sphingomonas | | 0.126 |
| 9ac3bb1d7dedb08a012692a6f536b5af | 4.1 | Alphaproteobacteria | Sphingomonas | | 0.474 |
| bcba47620c299fd34809f143e3d26dff | 3.7 | Alphaproteobacteria | Sphingomonas | | 0.233 |
| db66071d30f1e311d4458035a349d027 | 3.9 | Alphaproteobacteria | Sphingomonas | | 0.059 |
| e526b7bcb6bcc4067b9f0430c5399489 | 3.5 | Alphaproteobacteria | Sphingomonas | | 0.023 |
| fbdac2cf390ac4655eb3ff7f2de744d9 | 4.4 | Alphaproteobacteria | Sphingomonas | | 0.075 |
| ad16b779f2ec6d8afc200de7c5d2f39a | 4.0 | Alphaproteobacteria | Sphingopyxis | | 0.117 |
| 9c038c39cdaf030d74210a7b5a5f0734 | 3.6 | Alphaproteobacteria | Sphingorhabdus | | 0.024 |
| 1b3ab31378bec1762458af796e6f4b08 | 3.4 | Alphaproteobacteria | Unc. Sphingomonadaceae | | 0.027 |
| 45111cb138eeeecaf4eeffdf929707d8 | 3.8 | Alphaproteobacteria | Unc. Sphingomonadaceae | | 0.022 |
| ba7b5d03d6feb3245377d14103b5ce6e | 3.5 | Alphaproteobacteria | Unc. Sphingomonadaceae | | 0.068 |
| f23d11f758362cd09bfad7083fd86bbb | 4.4 | Alphaproteobacteria | Unc. Sphingomonadaceae | | 0.086 |
| 2672fd13538d2c0e313477a0424a749c | 3.7 | Alphaproteobacteria | Unc. Alphaproteobacteria | | 0.570 |
| 1d2c6bccd2a309860c56c2f5eee9e337 | 3.8 | Deltaproteobacteria | Unc. Blfdi19 | | 0.022 |
| 67355e95ba31971f190f204bb8274b04 | 3.4 | Deltaproteobacteria | Phaselicystis | | 0.043 |
| 7259b295d119e34a72b818f94bffe025 | 3.5 | Deltaproteobacteria | Pajaroellobacter | | 0.024 |
| a049257d7bbf044aecea70f98876eed5 | 3.5 | Deltaproteobacteria | Pajaroellobacter | | 0.024 |
| f37fc1dccfeba0004cbeb4f1ad6083c6 | 4.3 | Gammaproteobacteria | Duganella | | 0.402 |
| 4e6488dfc93f2eefe477c4511d0e8a49 | 4.1 | Gammaproteobacteria | Hydrogenophaga | | 0.169 |
| a21fc26b0d5f89da6136be69e1380e65 | 4.5 | Gammaproteobacteria | Hydrogenophaga | | 0.133 |
| 0a5a25f4502f341173aba7e91dd1a35a | 3.8 | Gammaproteobacteria | Ideonella | | 0.020 |
| d922e28fdfb36aa671e51df99219225a | 3.8 | Gammaproteobacteria | Leptothrix | | 0.164 |
| 3c9b3c34a5d4ff8b22d5e7cae520b7d0 | 3.5 | Gammaproteobacteria | Methylibium | | 0.618 |
| fa3ee069c33307053fe12d59a0d26f41 | 4.3 | Gammaproteobacteria | Methylibium | | 0.075 |
| c3357091b1eb1426ead2803433360b07 | 4.8 | Gammaproteobacteria | Noviherbaspirillum | | 0.261 |
| d4ecfe33623f297a74de51d7f28b39aa | 4.3 | Gammaproteobacteria | Pseudorhodoferax | | 0.190 |
| fc7b257064027e1bc2b6fbfb4584677d | 3.8 | Gammaproteobacteria | Rhizobacter | | 0.572 |
| 0054f49e4bd159d2b5d64c4a5e26135d | 4.1 | Gammaproteobacteria | Unc. Burkholderiaceae | | 0.046 |
| 01ef1f83c86823200c8c0e7597cd6a3a | 3.1 | Gammaproteobacteria | Unc. Burkholderiaceae | | 0.109 |
| 2b9972ea6484895a1a40f6b660ef1a8f | 3.7 | Gammaproteobacteria | Unc. Burkholderiaceae | | 0.045 |
| 3e6ed11dba3266973529e728a20bf957 | 4.4 | Gammaproteobacteria | Unc. Burkholderiaceae | | 0.436 |
| 47a9d10e32ffbceb163e240b65825d7e | 4.9 | Gammaproteobacteria | Unc. Burkholderiaceae | | 1.283 |
| 56aedcf6ae4ccd4e92f4a6e6429fe7be | 3.9 | Gammaproteobacteria | Unc. Burkholderiaceae | | 0.087 |
| 6abc517aa40e9e7b9c652902fe04bb1a | 4.4 | Gammaproteobacteria | Unc. Burkholderiaceae | | 0.259 |
| 6fd05e11fe57bb5ba9fd3b0fdd8b80f1 | 4.7 | Gammaproteobacteria | Unc. Burkholderiaceae | | 0.455 |
| 9f796d7ce01bf3256cb922eedbc7e0d1 | 4.2 | Gammaproteobacteria | Unc. Burkholderiaceae | | 0.054 |
| ba1561bbc611b63065c0eeb0f1197e14 | 4.1 | Gammaproteobacteria | Unc. Burkholderiaceae | | 0.047 |
| ba7c460bf2d0ff592fc2a6f198046b7b | 3.6 | Gammaproteobacteria | Unc. Burkholderiaceae | | 0.025 |
| d3530350480a2196e24a004fa0027558 | 4.6 | Gammaproteobacteria | Unc. Burkholderiaceae | | 0.323 |
| d590c23ecc6ae2ebb1cd5619aef7b97b | 3.9 | Gammaproteobacteria | Unc. Burkholderiaceae | | 0.027 |
| dc6a4cb8b307c33c3b87d1506423d4ed | 4.0 | Gammaproteobacteria | Unc. Burkholderiaceae | | 0.035 |
| f672aa3aea2d03f0b297739022e9b716 | 3.8 | Gammaproteobacteria | Unc. Burkholderiaceae | | 0.059 |
| f78576e24f12dd1e98e9df281c1ebbe7 | 4.4 | Gammaproteobacteria | Unc. Burkholderiaceae | | 0.299 |
| 1e104b993416995498346089f57fd3bf | 3.6 | Gammaproteobacteria | Methylophilus | | 0.074 |
| 31c51c7cfd98f27206b360d393dd2948 | 3.7 | Gammaproteobacteria | Methylotenera | | 0.030 |
| 731d9d7f7bce53a7dd5d190aabdedb5d | 4.2 | Gammaproteobacteria | Methylotenera | | 0.174 |
| dcaa56491d749ca0e1cbc91a2a3d3755 | 3.6 | Gammaproteobacteria | Unc. Methylophilaceae | | 0.043 |
| b7af60278d43e64c877784e4d885c00a | 3.3 | Gammaproteobacteria | Ellin6067 | | 0.035 |
| d6bbdaced0e23728c47239b6f83cafbd | 3.5 | Gammaproteobacteria | Ellin6067 | | 0.021 |
| 6112d362565c5797eb555106a550d353 | 3.7 | Gammaproteobacteria | Cellvibrio | | 0.025 |
| fd2899fba49792389b004ff5b4edc105 | 3.6 | Gammaproteobacteria | Cellvibrio | | 0.023 |
| 78fd02b88fb6af8c7d8f3f615935447f | 4.0 | Gammaproteobacteria | Aquicella | | 0.033 |
| c2435778f6ec07c2f8f824428e3fff56 | 3.7 | Gammaproteobacteria | Aquicella | | 0.025 |
| 945184b6386c192c0066e0a98a154780 | 3.9 | Gammaproteobacteria | Enterobacter | | 0.158 |
| 0f18144d308ada95632ab5193d92073f | 4.0 | Gammaproteobacteria | Pseudomonas | | 0.128 |
| 3abea7a304e461351b7de2cfc4b65846 | 3.5 | Gammaproteobacteria | Pseudomonas | | 0.024 |
| b4289ab0a9a00034c1fbce477b2c4ded | 4.0 | Gammaproteobacteria | Pseudomonas (Pseudomonas viridiflava) | | 0.104 |
| c8b9afe5f6282d1f00f655026543d8ec | 3.4 | Gammaproteobacteria | Unc. Steroidobacteraceae | | 0.020 |
| f36950a3d35495f3ee5aac81e18685f8 | 3.2 | Gammaproteobacteria | Dyella | | 0.181 |
| 8dddfea89ebf354cce15ed349857a3f5 | 3.7 | Gammaproteobacteria | Unc. Rhodanobacteraceae | | 0.041 |
| ae190110cb738b311ffd882ca15c4252 | 3.5 | Gammaproteobacteria | Unc. Rhodanobacteraceae | | 0.022 |
| 10c9c807090e88a746c9b282152fde70 | 3.5 | Gammaproteobacteria | Unc. Xanthomonadales | | 0.028 |
| c6ecf0fe8c87aa11b74dc6516b34b904 | 3.6 | Gammaproteobacteria | Arenimonas | | 0.040 |
| fc53f4c6401c6a2fb8190bdb3bb60648 | 3.7 | Gammaproteobacteria | Arenimonas | | 0.060 |
| e10a3c79a39afb01f516a753e612dd4c | 4.2 | Gammaproteobacteria | Luteimonas | | 0.056 |
| 7c286c1be50c5942270c41334d417c95 | 4.0 | Gammaproteobacteria | Pseudoxanthomonas | | 0.047 |
| b25042e177d93328dacdc1dbb769dab1 | 4.2 | Gammaproteobacteria | Pseudoxanthomonas | | 0.106 |
| f47beff0fc3748fcf424bf33bc490cf2 | 3.9 | Gammaproteobacteria | Pseudoxanthomonas | | 0.084 |
| 496ecde24f9ab698992413d3d4f04b5f | 3.9 | Gammaproteobacteria | Stenotrophomonas | | 0.056 |
| c2d5b1fe7e78d3d6f19b77c071c5c0f8 | 4.0 | Gammaproteobacteria | Xanthomonas (Xanthomonas campestris) | | 0.043 |
| 9bf08b3e87436850e71d2fb2bae207f1 | 3.3 | Verrucomicrobiae | Chthoniobacter | | 0.034 |
| ca3a23e08569cf875944dac81527e3c4 | 3.8 | Verrucomicrobiae | Chthoniobacter | | 0.024 |
| 3f81ddbf1e37df55c0b274186577654d | 3.8 | Verrucomicrobiae | Candidatus_Xiphinematobacter | | 0.023 |
| 6348e946199e8fa60d37ac2e765464c8 | 3.9 | Verrucomicrobiae | Lacunisphaera | | 0.028 |
| 57608fce970f7f3c1d7cacb6cd93cb1c | 3.6 | Verrucomicrobiae | Opitutus | | 0.034 |
| 042e74b5c8d47ba694ce24c2bed730b0 | 3.7 | Verrucomicrobiae | Luteolibacter | | 0.044 |
| 367fd0532f406448a26bcca3b54e8e2b | 3.9 | Verrucomicrobiae | Luteolibacter | | 0.070 |
| 3e1a26e59596ea4b27910d21a7f2685f | 3.7 | Verrucomicrobiae | Luteolibacter | | 0.103 |
| 0ad5811c815352c78b3a50978aad2edd | 3.7 | Verrucomicrobiae | Roseimicrobium | | 0.023 |
| 6bd8bdc08257c42a63097ae67d5590ff | 4.0 | Verrucomicrobiae | Unc. Verrucomicrobiaceae | | 0.172 |
| 69ea7bf023c94c0e9e322cf8722862e4 | 3.2 | Verrucomicrobiae | Verrucomicrobium | | 0.023 |
| 146195f72933e590d98b89c38d6eda33 | 3.4 | Actinobacteria | Geodermatophilus | | 0.031 |
| e918731f0def8c5e34473a87f277e62f | 3.5 | Armatimonadia | Unc. Armatimonadales | | 0.025 |
| cbcc718b54a5d4f76baf4aa2c7ffb097 | 3.7 | Bacteroidia | Ferruginibacter | | 0.077 |
| d9ea61005643a4051b118703705322ae | 3.7 | Bacteroidia | Ferruginibacter | | 0.041 |
| 8618e0050b4448db9314348748e44047 | 3.9 | Bacteroidia | Terrimonas | | 0.125 |
| 6348e24d925434a5aa8218148b5c279d | 4.1 | Bacteroidia | Unc. Chitinophagaceae | | 0.130 |
| 33dc047eea2341f7105bc0f78a193ca0 | 3.2 | Bacteroidia | Unc. Saprospiraceae | | 0.057 |
| 494db538559fffe9f1233e228cf3b4e0 | 3.5 | Bacteroidia | Cytophaga | | 0.031 |
| 0c4f8c95fedb27fdbdf839992673c20d | 3.8 | Bacteroidia | OLB12 | | 0.039 |
| 2a55261fb22634e1eeb49246ca99e55b | 4.2 | Bacteroidia | Unc. Microscillaceae | | 0.097 |
| 8c527e768c4215dde051230d503fc8f6 | 3.9 | Bacteroidia | Unc. Microscillaceae | | 0.063 |
| c7cf18b36e0a4630662c83970a30dfd4 | 3.9 | Bacteroidia | Unc. Microscillaceae | | 0.048 |
| e352928848235ea73d5ba4a4fbe4d748 | 3.8 | Bacteroidia | Unc. Microscillaceae | | 0.047 |
| 2b8313923596f09c18a4b6802c632b36 | 3.5 | Bacteroidia | Dyadobacter | | 0.032 |
| d93b123098236d27ea93bd41fa983e3b | 3.2 | Bacteroidia | Larkinella | | 0.029 |
| 061c698374f3607abb59bf33a925ef1d | 3.7 | Bacteroidia | Spirosoma | | 0.074 |
| d7c3c7544156a9d47929a449b0f58591 | 3.9 | Bacteroidia | Spirosoma | | 0.088 |
| 0dee873eac9bfa04eb672ce295ebbcee | 4.0 | Bacteroidia | Flavobacterium | | 0.064 |
| e61a7bc42a30f0ca4f170b5e3f6cad0c | 3.4 | Bacteroidia | Chryseobacterium | | 0.099 |
| 23620e81538019f85c727ec1791df244 | 3.7 | Bacteroidia | Mucilaginibacter | | 0.055 |
| 57806064e7b57eed3984469dc58f6140 | 3.5 | Bacteroidia | Mucilaginibacter | | 0.046 |
| 5a26205869ba51d709fd0f81a5fb12db | 4.5 | Bacteroidia | Mucilaginibacter | | 0.146 |
| ad0935af41c7af13338ce2d751488a3a | 4.4 | Bacteroidia | Mucilaginibacter | | 0.215 |
| b36b1ae2161ca5039ec88db9edb3c872 | 4.1 | Bacteroidia | Mucilaginibacter | | 0.062 |
| eded838989509e4970383c837ca72f01 | 3.9 | Bacteroidia | Mucilaginibacter | | 0.269 |
| 47e207d6c2becbf11ff6927edef8d9c4 | 3.8 | Chloroflexia | FFCH7168 | | 0.027 |
| f7f6db4376ca85aaa842a962597ff926 | 3.9 | Chloroflexia | FFCH7168 | | 0.100 |
| b47d251f99f4d7f5f043e781f720a4a7 | 3.8 | Chloroflexia | Unc. Chloroflexaceae | | 0.048 |
| ccadeb04c55b571c0885a7a915417e7f | 3.8 | Oxyphotobacteria | Phormidesmis_ANT.L52.6 | | 0.143 |
| 70411cc79cfd7029d493a37ef7c9b9ec | 4.3 | Oxyphotobacteria | Wilmottia_Ant-Ph58 | | 0.256 |
| 4aee044bee666c8984a0718a6ca2f3ec | 4.8 | Oxyphotobacteria | Tychonema_CCAP_1459-11B | | 1.152 |
| 123ebc98f5bb5ad6c4c76be377835bab | 5.2 | Oxyphotobacteria | Unc. Oxyphotobacteria_Incertae_Sedis | | 1.328 |
| 31b6e81120bc75aa74589615f84bc423 | 3.7 | Oxyphotobacteria | Unc. Oxyphotobacteria_Incertae_Sedis | | 0.047 |
| 4c2d3790830716f3b6ff0a198a19f46c | 3.6 | Oxyphotobacteria | Unc. Oxyphotobacteria_Incertae_Sedis | | 0.065 |
| e4d2ff134ed388b5711bae642db18036 | 3.6 | Gemmatimonadetes | Gemmatimonas | | 0.043 |
| 5271b01a0a9c7bd4575092999db6580d | 3.4 | Phycisphaerae | Unc. WD2101_soil_group | | 0.022 |
| 6638c7b6e8863db06d741eabc54ecd80 | 3.4 | Phycisphaerae | Unc. WD2101_soil_group | | 0.024 |
| 525cb4813e9d9ceb3771c27300f3be3c | 3.5 | Planctomycetacia | Gemmata | | 0.030 |
| 4a68df22f17735a23cb8eb6b7f113c68 | 3.9 | vadinHA49 | Unc. vadinHA49 | | 0.030 |
| 990af54737a612396bfcb924cfbbe72b | 3.3 | Alphaproteobacteria | Roseomonas | | 0.025 |
| c08751aa5c0ce81720da7543b8f64786 | 3.5 | Alphaproteobacteria | Unc. Acetobacteraceae | | 0.035 |
| a60f0b4bc20e5a162b45b3d2ed7a0d6c | 3.6 | Alphaproteobacteria | Caulobacter | | 0.060 |
| 2173ff99b55e25f3178d3943d534facc | 3.6 | Alphaproteobacteria | Phenylobacterium | | 0.027 |
| f120d43d6fcd4e8c2ff09268fcc41e4e | 3.7 | Alphaproteobacteria | Dongia | | 0.055 |
| e07417e82c2d3493a38ebd6765540920 | 3.3 | Alphaproteobacteria | Unc. Micavibrionales | | 0.029 |
| ca04af74472ae45465b2bf15ccf076ce | 3.7 | Alphaproteobacteria | alphaI_cluster | | 0.050 |
| 374b724806294b861c4ed5cfb74e2f31 | 4.0 | Alphaproteobacteria | Methylobacterium | | 0.159 |
| 397b2f8e42f9fa4f7e37216716ebfcfe | 3.4 | Alphaproteobacteria | Methylobacterium | | 0.021 |
| 7b9ce8e5c02aca74dd6c923413e99764 | 3.8 | Alphaproteobacteria | Methylobacterium | | 0.059 |
| 7e8d4aa8fe2e2253d3aba3e515ca2c51 | 4.2 | Alphaproteobacteria | Allorhizobium-Neorhizobium-Pararhizobium-Rhizobium | | 0.095 |
| 8a83da895f30577482440adf41ccfcf0 | 3.9 | Alphaproteobacteria | Allorhizobium-Neorhizobium-Pararhizobium-Rhizobium | | 0.129 |
| a9164d01308501b576afa06256a15b0f | 4.5 | Alphaproteobacteria | Allorhizobium-Neorhizobium-Pararhizobium-Rhizobium | | 0.244 |
| 41da1f67db882e54aa2020701805031e | 4.0 | Alphaproteobacteria | Rhodopseudomonas | | 0.155 |
| 83bfcfd2290b7c4261e0cbdcc96c0b97 | 4.0 | Alphaproteobacteria | Unc. Rhodobacteraceae | | 0.128 |
| b56c9d82534f9b8b62c40c2e6f267bb7 | 3.7 | Alphaproteobacteria | Novosphingobium | | 0.061 |
| 98a97c8c238eded7aa70ac0af471c46f | 3.9 | Alphaproteobacteria | Rhizorhapis | | 0.128 |
| 44349931377434a54bb432488ce41d8f | 4.0 | Alphaproteobacteria | Sphingomonas | | 0.085 |
| 02d37f45629c4adfae251010a6b7c1ab | 3.5 | Alphaproteobacteria | Unc. Alphaproteobacteria | | 0.051 |
| 975dad73b6b5c8a4f3170049d1c88b43 | 4.0 | Gammaproteobacteria | Acidovorax | | 0.144 |
| cc3cdd2ff9769ffe23e1c9cb097e0af8 | 4.1 | Gammaproteobacteria | Herbaspirillum | | 0.056 |
| f721d83108dd078d9769b367dfa3c1d9 | 4.5 | Gammaproteobacteria | Massilia | | 0.273 |
| 25cb02fb17426d878afd69a79572b858 | 4.0 | Gammaproteobacteria | Polaromonas | | 0.199 |
| e8724520788bb38a7f9d22c91318f016 | 4.2 | Gammaproteobacteria | Rhizobacter | | 0.573 |
| aed191bac0b1c3264208a084af848bed | 3.9 | Gammaproteobacteria | Rhodoferax | | 0.518 |
| f34cdcd73e535507d29c29b19d01f658 | 3.7 | Gammaproteobacteria | Rhodoferax | | 0.109 |
| e416d0916760d2fc17b616e2ac3ad855 | 3.8 | Gammaproteobacteria | Methylotenera | | 0.041 |
| cc761daf51f27c423da57f3f1f0ff5cc | 3.9 | Gammaproteobacteria | Pantoea | | 0.039 |
| 0ddcd311e02f742e2e0e61ce02cf9c29 | 4.3 | Gammaproteobacteria | Pseudomonas | | 0.137 |
| 9f775e92b88a1437af90dc4a544e7a5f | 3.6 | Gammaproteobacteria | Luteibacter | | 0.020 |
| 14d05bcb6de5774c6a83affe499761f1 | 3.9 | Gammaproteobacteria | Thermomonas | | 0.087 |
| 1709eee19967e40cdfc7154f64dce114 | 3.7 | Verrucomicrobiae | Chthoniobacter | | 0.061 |
| a6e1599ff40db75eaf7214a826be285c | 3.9 | Verrucomicrobiae | Chthoniobacter | | 0.048 |
| 19bf0c1b9f4d2805e5df22736695b103 | 4.0 | Verrucomicrobiae | Prosthecobacter | | 0.074 |
| 1a76b31651a5561f9002b1096c1a426b | 3.5 | Verrucomicrobiae | Roseimicrobium | | 0.024 |
| 62f7ecf7d05f92813a3f21b862040dda | 4.0 | Verrucomicrobiae | Verrucomicrobium | | 0.085 |
| 5011a0ea9d7402163345550ceb728e0d | 3.5 | Unc. WPS-2 | Unc. WPS-2 | | 0.022 |
| 8562afaf3955423d4eda6d71b8b277d8 | 3.9 | Blastocatellia_(Subgroup_4) | RB41 | | 0.061 |
| a831520d4ccdc381bf81d5733699ddfd | 3.5 | Armatimonadia | Unc. Armatimonadales | | 0.036 |
| a5aa30da0b2685c7b0c47be160249bee | 3.4 | Bacteroidia | Flavitalea | | 0.020 |
| a8df653da66acd4d326cda1667bc2941 | 3.5 | Bacteroidia | Lacibacter | | 0.060 |
| ed45fc478ac528cb6c53d5188897ddf7 | 3.8 | Bacteroidia | Unc. Saprospiraceae | | 0.036 |
| 10653b7a7516a0d43c68e9b8bb771a8f | 3.4 | Bacteroidia | Chryseolinea | | 0.024 |
| fb0b99a33771ab37c61cccd46891f54a | 3.3 | Bacteroidia | Unc. Microscillaceae | | 0.045 |
| d15bd1dcb9de71d7452648673a59f5b8 | 3.8 | Bacteroidia | Pedobacter | | 0.271 |
| b6a7369fb4bf1be14116033b76a0af4f | 3.8 | Chloroflexia | FFCH7168 | | 0.038 |
| aa18e1c2f6149435a333e1cf128b27c5 | 4.2 | Chloroflexia | Unc. Chloroflexaceae | | 0.244 |
| 111e022a19c811e209e03999c398f113 | 3.5 | Oxyphotobacteria | Unc. Leptolyngbyaceae | | 0.029 |
| f3e8b73fc6a41d5dbf5ff87857f191b3 | 5.5 | Oxyphotobacteria | Unc. Coleofasciculaceae | | 5.290 |
| 6ad66b90bca7ea98c7dd6eb6dfc74657 | 4.7 | Oxyphotobacteria | Nostoc_PCC-73102 | | 0.421 |
| 6033d3285cd5401519784fc3709ffa9d | 4.0 | Oxyphotobacteria | Unc. Oxyphotobacteria_Incertae_Sedis | | 0.192 |
| eaad01515fc39f898a5afa7883d6a246 | 3.8 | Oxyphotobacteria | Unc. Oxyphotobacteria_Incertae_Sedis | | 0.228 |
| 117dea1a2ad5db7890155aa5e018e9c3 | 4.1 | Oxyphotobacteria | Nodosilinea_PCC-7104 | | 0.171 |
| 2dc7087fc87a98349d880c5cc9b29f49 | 3.4 | Alphaproteobacteria | Phenylobacterium | | 0.042 |
| 455cee7813d5211906cd43c371990c3d | 4.1 | Alphaproteobacteria | Unc. Caulobacteraceae | | 0.037 |
| 3209a1e49860e30a9135e714f413fd15 | 3.7 | Alphaproteobacteria | Unc. Rhizobiales_Incertae_Sedis | | 0.077 |
| f4089dbf9f29b31de4628aca91af0ebe | 3.7 | Alphaproteobacteria | Unc. Rhizobiales_Incertae_Sedis | | 0.036 |
| 8495a7602793efbeca7e9822804d198a | 3.9 | Alphaproteobacteria | Unc. Rhodobacteraceae | | 0.026 |
| 312562ce33611e742b6e57c7d754fbe0 | 4.3 | Alphaproteobacteria | Novosphingobium | | 0.107 |
| 1e2f6526aade1b144f211784b2698e81 | 3.5 | Alphaproteobacteria | Sphingoaurantiacus | | 0.028 |
| cd0ff809ace0c955b41b32a8e16883e3 | 3.5 | Alphaproteobacteria | Sphingomonas | | 0.040 |
| 655273c1c38e976754fa517fa9a40f4a | 4.1 | Gammaproteobacteria | Unc. Burkholderiaceae | | 0.229 |
| 7d2c0bf77d1048b740a0eb47b3f80c23 | 4.0 | Gammaproteobacteria | Unc. Burkholderiaceae | | 0.204 |
| 3d67411a8df43090196f48b82ee031d1 | 3.3 | Gammaproteobacteria | Unc. Gammaproteobacteria_Incertae_Sedis | | 0.021 |
| 6376ea6dab7d0fde3cd66f53b57e1484 | 4.3 | Gammaproteobacteria | Pseudomonas | | 0.487 |
| c06ccd1f7d57566ef669942328b1a946 | 3.9 | Gammaproteobacteria | Pseudomonas | | 0.167 |
| 8385345dad8cab2b5f25cdd7455e67a6 | 3.9 | Verrucomicrobiae | Candidatus_Xiphinematobacter | | 0.024 |
| cc52d51ef41eda377f6f26631bdaa7b7 | 3.8 | Verrucomicrobiae | Opitutus | | 0.097 |
| e24b8fd23804052c8a536f242997d7dd | 3.4 | Verrucomicrobiae | Unc. Verrucomicrobiaceae | | 0.030 |
| 1d6b9da3bd2d4ac1a99d1a57a422c3f1 | 3.7 | Nitrososphaeria | Unc. Nitrososphaeraceae | | 0.052 |
| a3fe6144bb655c43f48abfa8bc92709d | 3.7 | Nitrososphaeria | Unc. Nitrososphaeraceae | | 0.051 |
| b590eb8bb692cc36076c9cadb6a10e79 | 3.6 | Nitrososphaeria | Unc. Nitrososphaeraceae | | 0.028 |
| f0ccfc9bb93021c9fc6b4acffb404afc | 3.8 | Nitrososphaeria | Unc. Nitrososphaeraceae | | 0.046 |
| ceb36da1a26eecc6429aa5cefb368a09 | 3.2 | Acidobacteriia | Unc. Acidobacteriales | | 0.049 |
| d7d5ad3fd42fd1ff7918613de090cf05 | 3.8 | Acidobacteriia | Bryobacter | | 0.041 |
| edb9c10a487ad4ace7c08ebe26d7145a | 3.7 | Acidobacteriia | Bryobacter | | 0.032 |
| f4e02ec0242b8d3731f2eb480154c315 | 3.6 | Acidobacteriia | Bryobacter | | 0.065 |
| 1547e1c203c67b773f73c1fef1072566 | 3.5 | Blastocatellia_(Subgroup_4) | JGI_0001001-H03 | | 0.074 |
| ef98cc2984cd3ce0ca6a46589a6df194 | 4.2 | Blastocatellia_(Subgroup_4) | JGI_0001001-H03 | | 0.188 |
| e6ae687fa14ab2cd9955578eb02bdcab | 3.8 | Blastocatellia_(Subgroup_4) | RB41 | | 0.060 |
| 8af9b72c1b3847562719d980e6cab6ef | 3.6 | Subgroup_6 | Unc. Subgroup_6 | | 0.029 |
| ad145b5c8b7c9b7ffb1c647fa0da640d | 4.0 | Subgroup_6 | Unc. Subgroup_6 | | 0.176 |
| 1975f0f2b6531e29f1c84e99f1b51ef8 | 3.3 | Actinobacteria | Acidothermus | | 0.054 |
| fe0b139ead8552cf0813868bc8fc75c7 | 3.8 | Actinobacteria | Phycicoccus | | 0.067 |
| b17a2f19f4c913ea1af58c2cf5b3abbe | 3.5 | Actinobacteria | Terrabacter | | 0.060 |
| fc4d567d1d0828e1fdc36916f0d53d15 | 3.3 | Actinobacteria | Actinoplanes | | 0.025 |
| 68c56896f754db932a51a88ebad5bc6a | 3.5 | Actinobacteria | Nocardioides | | 0.036 |
| a5e48b027fcf233fa5c9a33e67ddd2c4 | 3.4 | Thermoleophilia | Gaiella | | 0.023 |
| 86e14dd85367bf944f87fb172a7697ea | 3.6 | Thermoleophilia | Unc. Gaiellales | | 0.051 |
| d66ae513dcd054596701af5cfd106aed | 3.4 | Armatimonadia | Unc. Armatimonadales | | 0.020 |
| 2dcef2e3399d94ba6ac2a0f2f9f31c4c | 4.4 | Bacteroidia | Ferruginibacter | | 0.146 |
| 6ca072aeb676074a4f3eeda5740f38be | 4.2 | Bacteroidia | Ferruginibacter | | 0.088 |
| cc92e59ca899dbd31fe97f6344e82ba9 | 4.0 | Bacteroidia | Ferruginibacter | | 0.056 |
| d798484e097b6518f50a27ccf383ca37 | 4.1 | Bacteroidia | Ferruginibacter | | 0.045 |
| dfaf9cbc71eda99adf1bb93cc4be9a07 | 3.6 | Bacteroidia | Ferruginibacter | | 0.032 |
| e1ed6b8bc192db07db757992cf49470f | 3.7 | Bacteroidia | Ferruginibacter | | 0.034 |
| 67aa187e7915900cc6979df994fc867c | 3.5 | Bacteroidia | Flavihumibacter | | 0.026 |
| 69b3be6879a865e22dcfae668de16708 | 3.4 | Bacteroidia | Flavisolibacter | | 0.046 |
| 3a243c419f242f78d33de701e1c590d0 | 3.6 | Bacteroidia | Parasegetibacter | | 0.061 |
| 13fd47213c2ac0a5bc2776572e8cbd88 | 3.4 | Bacteroidia | Terrimonas | | 0.048 |
| 85719161db021464513f7c3d5a3d02eb | 3.8 | Bacteroidia | Unc. Chitinophagaceae | | 0.040 |
| af7da67e9f7eda1e214f26eaa9ac9d55 | 3.6 | Bacteroidia | Unc. Chitinophagaceae | | 0.071 |
| 07fa00f734b717f43bd95dbca5224947 | 3.4 | Bacteroidia | Candidatus_Amoebophilus | | 0.037 |
| e60021903181c4747e9a6ec7eb85565f | 3.4 | Bacteroidia | Unc. Microscillaceae | | 0.021 |
| f617f0ce888fe9b6b91cb9069b7a11f5 | 4.1 | Bacteroidia | Dyadobacter | | 0.064 |
| 2ca2d98e0489dc0314d8c79541be9115 | 4.7 | Bacteroidia | Emticicia | | 0.221 |
| cc0f02e357468fe01879f0c91c5edffa | 4.4 | Bacteroidia | Flavobacterium | | 0.122 |
| dddb6d460c953d92bf9a3eee51155857 | 3.8 | Bacteroidia | Flavobacterium | | 0.022 |
| 19c8643ae0215cd6647a6482dafc05c2 | 4.0 | Bacteroidia | Mucilaginibacter | | 0.147 |
| 78fd66d99d6b013e4426236a89124d6a | 4.0 | Bacteroidia | Mucilaginibacter | | 0.327 |
| a753f742930e6d35d887a334bebfc461 | 4.5 | Bacteroidia | Mucilaginibacter | | 0.116 |
| 4ec3e45b26626e6f79c68bb9861d9aa1 | 3.9 | Anaerolineae | Unc. A4b | | 0.027 |
| b090a09cec21542703d779fa6a2479b9 | 3.8 | Anaerolineae | Unc. A4b | | 0.034 |
| b3c40552fec4d0b1f6cc4c7bbf6496fa | 3.9 | Anaerolineae | Unc. A4b | | 0.041 |
| 6631126838cb98ec2800beebac288be4 | 3.4 | Chloroflexia | FFCH7168 | | 0.031 |
| 9fc2c06c798d6f4ec0b2913916160007 | 4.3 | Chloroflexia | FFCH7168 | | 0.189 |
| e04dc9f11a0b336f93b31a7b43211b94 | 3.9 | Chloroflexia | FFCH7168 | | 0.093 |
| c3709f6d60f200e02e4a239cf622b38c | 3.6 | Chloroflexia | Unc. Chloroflexaceae | | 0.042 |
| 111e8e48a5111d4360329eef12acd3b7 | 3.5 | Chloroflexia | Unc. Roseiflexaceae | | 0.024 |
| d899e645f1d8a9d189a96277f0a5a81a | 3.8 | Chloroflexia | Unc. Roseiflexaceae | | 0.056 |
| ebb0349f3f4010c2cf9c3bffc4f5061e | 3.2 | Chloroflexia | Unc. JG30-KF-CM45 | | 0.025 |
| f4be2f08546d37a21f58a82ff64fa989 | 3.3 | Chloroflexia | Unc. JG30-KF-CM45 | | 0.032 |
| c710e81ef0729edbd6e9f9899b2b4677 | 3.4 | Ktedonobacteria | Unc. C0119 | | 0.024 |
| 46cc5948892ea0997452f8280123454c | 2.9 | Oxyphotobacteria | Unc. Leptolyngbyaceae | | 0.180 |
| 8bdc7d4f7384a16862f4d1063270d14d | 4.3 | Oxyphotobacteria | Unc. Leptolyngbyaceae | | 0.233 |
| 1ad9e23d708017c0783a416a3a35a6bf | 3.2 | Oxyphotobacteria | Unc. Coleofasciculaceae | | 0.259 |
| 71cae704631791daeb8c616421d59652 | 2.9 | Oxyphotobacteria | Unc. Coleofasciculaceae | | 0.099 |
| b5b8e22b2e406e3a6d3484caa445f4a4 | 4.4 | Oxyphotobacteria | Unc. Coleofasciculaceae | | 0.856 |
| ac44246b11ca5893bb2d215c0cf09206 | 4.3 | Oxyphotobacteria | Nostoc_PCC-73102 | | 0.095 |
| eb96d3a3bbcdd829a48a49f77de2f98f | 4.5 | Oxyphotobacteria | Nostoc_PCC-73102 | | 0.588 |
| 1f628831b1709d15c1799e5a5e0cc084 | 3.6 | Oxyphotobacteria | Unc. Nostocaceae | | 0.034 |
| 5fd1c26b10700dc41be4d29fd14842ca | 3.8 | Oxyphotobacteria | Unc. Nostocaceae | | 0.021 |
| 98ebd885852f9c108b13365084c2f067 | 4.2 | Oxyphotobacteria | Unc. Nostocaceae | | 0.229 |
| 9fcad870defe3d376932305565ce2a16 | 3.2 | Oxyphotobacteria | Unc. Nostocaceae | | 0.336 |
| db56f5915628e529335c0f15b845d5ee | 4.1 | Oxyphotobacteria | Unc. Nostocaceae | | 0.058 |
| e58415f192423183f67e39502b46bd45 | 5.1 | Oxyphotobacteria | Unc. Nostocaceae | | 1.371 |
| 817614455305017b9e38c74918681cbe | 4.3 | Oxyphotobacteria | Unc. Oxyphotobacteria_Incertae_Sedis | | 0.173 |
| be71f34324cb0beaf6a7b275e8cb349f | 2.9 | Oxyphotobacteria | Unc. Oxyphotobacteria_Incertae_Sedis | | 0.328 |
| 68ca776355d44c624ea54eae0bb04ca6 | 2.7 | Oxyphotobacteria | Unc. Pseudanabaenaceae | | 0.136 |
| 6c5f4f9666d82b1bedf94e99acfe6741 | 4.2 | Bacilli | Bacillus | | 0.267 |
| 83e948f389f9d97b0c975afc880989e8 | 3.7 | Bacilli | Bacillus | | 0.089 |
| 2d9a9d6012e671d8135cb2df734d9edd | 2.8 | Gemmatimonadetes | Gemmatimonas | | 0.073 |
| 3501d03f4da902cab194448b80dbcb86 | 3.4 | Gemmatimonadetes | Gemmatimonas | | 0.039 |
| 214a9805d0ba28f1966432d4ac7e884d | 3.5 | Planctomycetacia | Unc. Pirellulaceae | | 0.031 |
| d3d472c89611f2b47bbe1b12747001e8 | 3.4 | Alphaproteobacteria | Unc. Azospirillales | | 0.024 |
| e529f921adcae0ca6ba1d9148d23802a | 3.4 | Alphaproteobacteria | Phenylobacterium | | 0.022 |
| fcacc1a798bb0322e8cd110c6d8d1011 | 3.8 | Alphaproteobacteria | Unc. Micropepsaceae | | 0.082 |
| 4fa9023e0e34aa50c180c60440bfb741 | 3.1 | Alphaproteobacteria | Unc. A0839 | | 0.093 |
| 1fd1f3fbc0437b678ede5a76465a1326 | 4.0 | Alphaproteobacteria | alphaI_cluster | | 0.163 |
| 1c2b8892a0d7f828d72808cd01cfd199 | 4.0 | Alphaproteobacteria | Devosia | | 0.365 |
| c130baecd35f08a8f79bdfbdc0abb6c0 | 4.0 | Alphaproteobacteria | Allorhizobium-Neorhizobium-Pararhizobium-Rhizobium | | 0.209 |
| 2988fa1676e6fdd0af3bf6ba655a3569 | 3.5 | Alphaproteobacteria | Unc. Rhizobiales_Incertae_Sedis | | 0.030 |
| 3a7bcfd3dd1c23c2efae4b6b3fcd36a3 | 3.6 | Alphaproteobacteria | Bradyrhizobium | | 0.044 |
| 6472eb8b1e09f892aca2f23182962903 | 3.8 | Alphaproteobacteria | Bradyrhizobium | | 0.084 |
| d829bee4984f82ffc2453212157caf96 | 4.1 | Alphaproteobacteria | Bradyrhizobium | | 0.646 |
| c63a29416a7eabd7ff67a49489118771 | 3.3 | Alphaproteobacteria | Unc. Xanthobacteraceae | | 0.031 |
| 481de4037ed3f3bcf51b47e548dcbd9e | 3.6 | Alphaproteobacteria | Novosphingobium | | 0.021 |
| d6efd2da2728fd74ded268122ee05036 | 3.9 | Alphaproteobacteria | Sphingomonas | | 0.065 |
| e71c86754bc9e5d7e0aa0f297bd99dda | 3.8 | Alphaproteobacteria | Sphingomonas | | 0.034 |
| 0032ab5da12fadd744023dc9bbba5169 | 3.5 | Gammaproteobacteria | Unc. A21b | | 0.024 |
| b760d597e018a73c8f6ff59001f23f0b | 4.1 | Gammaproteobacteria | AAP99 | | 0.129 |
| 12f0627b0f930aff1182481eb84cbc7d | 4.2 | Gammaproteobacteria | Aquabacterium | | 0.088 |
| 4608fae4bbc9964cdd17af8782f2155e | 4.7 | Gammaproteobacteria | Massilia | | 0.426 |
| ca6c2b3b469c08212142e8821c65882a | 3.9 | Gammaproteobacteria | Massilia | | 0.054 |
| f4801b7a68515d9005fa572ee6afdf41 | 3.3 | Gammaproteobacteria | Ralstonia (Ralstonia syzygii subsp. syzygii) | | 0.039 |
| 9dcc5f01b6693e2f5cd147e0427e7beb | 3.9 | Gammaproteobacteria | Rhodoferax | | 0.062 |
| 584dbd9cf4b609aecf37caf15ee9ebab | 4.2 | Gammaproteobacteria | Unc. Burkholderiaceae | | 0.080 |
| 585acbab6a2210c0f4feb3a5180a89a3 | 3.6 | Gammaproteobacteria | Unc. Burkholderiaceae | | 0.515 |
| 9c5a08f7a567648f9428c111774c2260 | 4.7 | Gammaproteobacteria | Unc. Burkholderiaceae | | 0.302 |
| ce0932e3265dfbbdb1378bb64b6cc5b2 | 3.8 | Gammaproteobacteria | Unc. Burkholderiaceae | | 0.036 |
| a843fc211658b32d18ca8567f97f1a1e | 3.9 | Gammaproteobacteria | Variovorax | | 0.045 |
| bf1969c0e412e57637a9ae7a3b638492 | 3.9 | Gammaproteobacteria | Variovorax | | 0.043 |
| facf0a18c6b8cfb59f661168c07f1e03 | 4.1 | Gammaproteobacteria | Methylotenera | | 0.107 |
| d381b0c4baf4c092e37d645b910d42d7 | 3.9 | Gammaproteobacteria | Ellin6067 | | 0.055 |
| ed4eb0c93690ed93b1306bbc507ef2ba | 3.8 | Gammaproteobacteria | mle1-7 | | 0.063 |
| 332864e3168e91e2d124d018a0f71974 | 3.7 | Gammaproteobacteria | Unc. SC-I-84 | | 0.029 |
| 4027a1857865117ca02f6277aa2b37ac | 3.6 | Gammaproteobacteria | Unc. SC-I-84 | | 0.083 |
| 6e6f306a337ba255a7f4a7f8469ff691 | 4.3 | Gammaproteobacteria | Cellvibrio | | 0.122 |
| 86d6db0b6e15510fa5d27ceae8fd5e0d | 3.7 | Gammaproteobacteria | Pseudomonas | | 0.042 |
| 46edd9a681a1c394007859666f76d477 | 3.6 | Gammaproteobacteria | Luteibacter | | 0.136 |
| 0af2aee8c0040db1b011087cf18f51cc | 3.6 | Gammaproteobacteria | Arenimonas | | 0.051 |
| b60765bf2e10d524fe9872a961bc4c53 | 3.9 | Gammaproteobacteria | Arenimonas | | 0.072 |
| d5597224e77808aa34962e88365b21da | 4.1 | Gammaproteobacteria | Arenimonas | | 0.045 |
| 69023e9c72ccf5e979a500bf1c235026 | 3.5 | Verrucomicrobiae | Candidatus_Udaeobacter | | 0.041 |
| 8abdf967e5b46a4af2dc34923219a392 | 4.0 | Verrucomicrobiae | Candidatus_Udaeobacter | | 0.202 |
| 9e04003825b786bb57df86bd48e6b9f8 | 3.7 | Verrucomicrobiae | Candidatus_Udaeobacter | | 0.059 |
| b0546a8c6a38415c52d2dc916319d3fd | 3.8 | Verrucomicrobiae | Candidatus_Udaeobacter | | 0.234 |
| 06201419387b9de1d0cfd85df261347b | 3.7 | Verrucomicrobiae | Chthoniobacter | | 0.034 |
| 21d0385c2e94cbc178fc8986b1688f79 | 3.8 | Verrucomicrobiae | Chthoniobacter | | 0.030 |
| 9169bf917c18782d253cd25c935164ca | 3.4 | Verrucomicrobiae | Chthoniobacter | | 0.025 |
| f6ef60c91b599d120920ede216b6af8e | 3.4 | Verrucomicrobiae | Chthoniobacter | | 0.046 |
| 7827d172fdf2185978863eb87542dd41 | 3.7 | Verrucomicrobiae | Roseimicrobium | | 0.036 |
| e921e545e3e42c795f12a215f520b1e2 | 4.2 | Blastocatellia_(Subgroup_4) | Aridibacter | | 0.104 |
| 48a2fef2d869ef51bd9c82f5017c72dd | 3.9 | Thermoanaerobaculia | Subgroup_10 | | 0.041 |
| a3d7ab745b47e86ab463b688dcec88b3 | 3.7 | Armatimonadia | Unc. Armatimonadales | | 0.027 |
| 0e9ae3d0137092a4fbf8d113097c38af | 4.0 | Bacteroidia | Flavisolibacter | | 0.042 |
| 683d7ebfecfa72db7fc109d35c397914 | 4.2 | Bacteroidia | Unc. Chitinophagaceae | | 0.081 |
| d5a5c8f530423b6fc84d830e90ac433b | 3.6 | Bacteroidia | Unc. Chitinophagaceae | | 0.062 |
| 286d57e898ad1cb1f5388dd351e521b5 | 3.8 | Bacteroidia | Flavobacterium | | 0.058 |
| 896e7d78ea2659016cfa15853fbbdd4a | 4.4 | Bacteroidia | Mucilaginibacter | | 0.210 |
| 177681e107264958ff375252377883f9 | 3.9 | Chloroflexia | FFCH7168 | | 0.042 |
| 56930f9f8316ad0bba7049826d057111 | 3.6 | Chloroflexia | FFCH7168 | | 0.023 |
| bf5ba2a740a713183ca18c097ac736e8 | 4.3 | Chloroflexia | FFCH7168 | | 0.172 |
| a933340217d5525b38d864498e7a53c7 | 4.1 | Oxyphotobacteria | Phormidesmis_ANT.L52.6 | | 0.077 |
| 4ccce8bf47f920ce2b2c2e08e38b2db9 | 4.6 | Oxyphotobacteria | Unc. Coleofasciculaceae | | 0.130 |
| a8fc8720c6451a36643c950bc5dbb832 | 4.2 | Oxyphotobacteria | Nostoc_PCC-73102 | | 0.074 |
| e49982af31b379ea48df231c15f8fc73 | 3.6 | Oxyphotobacteria | Tolypothrix_PCC-7601 | | 0.054 |
| 9a4c38a6ba457dc97a9aa32b37f9db88 | 4.4 | Oxyphotobacteria | Unc. Nostocales | | 0.080 |
| 1eeaaeccfd472abab3c3c038e87563f0 | 3.8 | Oxyphotobacteria | Unc. Oxyphotobacteria_Incertae_Sedis | | 0.036 |
| 7b505be6f098dc9ab5b100177f9006d1 | 3.1 | Oxyphotobacteria | Unc. Oxyphotobacteria_Incertae_Sedis | | 0.029 |
| 88703d1758d8974e903c91e576219fa0 | 4.1 | Oxyphotobacteria | Unc. Oxyphotobacteria_Incertae_Sedis | | 0.047 |
| a137d2ed9aaef41cebb43ecf91f7794e | 4.1 | Oxyphotobacteria | Unc. Oxyphotobacteria_Incertae_Sedis | | 0.055 |
| fdaadf57687444e1787bf2b72b5749f8 | 4.3 | Oxyphotobacteria | Unc. Oxyphotobacteria_Incertae_Sedis | | 0.095 |
| 402f7b5a9279452dd51c413b3de5b85d | 2.9 | Oxyphotobacteria | Unc. Oxyphotobacteria | | 0.126 |
| 5b707538be256f1ac3e23b60bbe2b22d | 3.9 | Clostridia | Unc. Clostridia | | 0.076 |
| 35e48064d71b163be36ce2ac9ba1b2ae | 4.1 | Phycisphaerae | Unc. WD2101_soil_group | | 0.058 |
| ee6b2a4b31df092b72c64a13d54575d5 | 3.6 | Phycisphaerae | Unc. WD2101_soil_group | | 0.024 |
| 92f1720367db58c68a96eceb9feb416a | 3.5 | Alphaproteobacteria | Methylobacterium | | 0.022 |
| 928b67e64faa0e5503b45c9c643bdcdc | 3.7 | Alphaproteobacteria | Rubellimicrobium | | 0.029 |
| 3d6da4853055ccfc80db03cefee88f5a | 4.1 | Alphaproteobacteria | Sphingomonas | | 0.105 |
| 50b61496d772ef01f3cbdacc5c6ca371 | 4.0 | Alphaproteobacteria | Sphingomonas | | 0.049 |
| 67b44c397cdc30646117216f1f794b6a | 3.7 | Alphaproteobacteria | Sphingomonas | | 0.040 |
| 832285aae93ea2d7c8bb1010f6809539 | 3.8 | Alphaproteobacteria | Sphingomonas | | 0.047 |
| c70d023fe6256e1a2d03c1dc62713ca1 | 4.2 | Alphaproteobacteria | Sphingomonas | | 0.076 |
| 0697aa6efec5a3702949fd86c35b6c9b | 3.7 | Alphaproteobacteria | Unc. Sphingomonadaceae | | 0.034 |
| fed24691259ad53bae4ae128168a1870 | 3.6 | Alphaproteobacteria | Unc. Sphingomonadaceae | | 0.035 |
| ef842304c4abd41a0d12022583c3e8be | 3.9 | Gammaproteobacteria | Hydrogenophaga | | 0.094 |
| c6e9c9fe9159441c4ffe44947875236a | 3.8 | Gammaproteobacteria | Rhizobacter | | 0.078 |
| 8012da7c810e58147175f741af2a78d2 | 4.4 | Gammaproteobacteria | Unc. Burkholderiaceae | | 0.198 |
| f36c50279a8fa1c121cb9dda1ab1cb42 | 4.2 | Gammaproteobacteria | Unc. Burkholderiaceae | | 0.249 |
| df71de0e92459aa808364311135bb8fc | 3.6 | Gammaproteobacteria | Variovorax | | 0.104 |

**Supplementary Table S3.** List of fungal ASVs that were significantly enriched in thallus samples in comparison to the surrounding soil according to linear discriminant analysis effect size (LEfSe) analysis and their ecological guild according to FUNGuild

| ASV ID | Taxonomical information | | Relative abundance (%) | Ecological guild |
| --- | --- | --- | --- | --- |
|  | Class | Genus |  |  |
| 31710f04c1d3def7bfea4ec1a43757f0 | Dothideomycetes | Cladosporium | 0.276 | na |
| 06fe039e6c439c062fea71deda5adb84 | Dothideomycetes | Unc._Didymellaceae | 0.08 | Animal Pathogen-Plant Pathogen-Undefined Saprotroph |
| 3579bd9a7985dc76cb4d728f2bc3b291 | Dothideomycetes | Unc._Didymellaceae | 0.734 | Animal Pathogen-Plant Pathogen-Undefined Saprotroph |
| bf5b3d89020b717ed5a86a0ad6f1c568 | Dothideomycetes | Unc._Didymellaceae | 5.846 | Animal Pathogen-Plant Pathogen-Undefined Saprotroph |
| ccfab76388bc41a39c02f86215622a0a | Dothideomycetes | Unc._Didymellaceae | 0.035 | Animal Pathogen-Plant Pathogen-Undefined Saprotroph |
| 500fd43697a5b0eade33dafbdc97b439 | Dothideomycetes | Unc._Phaeosphaeriaceae | 0.193 | Fungal Parasite-Plant Pathogen-Plant Saprotroph |
| 6ce7604e712f963bbd0ae0f6b7a3cf5e | Dothideomycetes | Alternaria | 1.154 | Animal Pathogen-Endophyte-Plant Pathogen-Wood Saprotroph |
| 20cb2381c7329d2363cd71598db48a88 | Dothideomycetes | Unc._Pleosporales | 0.273 | na |
| abd6c202e5d0874b9c43ba9ff0a3fff2 | Eurotiomycetes | Rhinocladiella | 0.031 | Undefined Saprotroph-Wood Saprotroph |
| 3fe9dcd9a20fe6e5fef54533f925c59d | Leotiomycetes | Hymenoscyphus | 0.025 | Bryophyte Parasite-Ectomycorrhizal-Ericoid Mycorrhizal-Undefined Saprotroph |
| f3fd364007b89f16f8b252f73daa3d9c | Leotiomycetes | Unc._Helotiales | 0.034 | na |
| baf7b8771974e996dbe2ad60d1cc7216 | Sordariomycetes | Unc. Sordariomycetes | 0.051 | na |
| 7fbb93d82e4abea98be2b43aa3e311fd | Unc. Ascomycota | Unc. Ascomycota | 3.38 | na |
| 4674f4ee98aa79fee390da5c7d1ccb6b | Agaricomycetes | Psathyrella | 0.039 | Wood Saprotroph |
| 52a21c107edd8df09a971fa76af060bb | Agaricomycetes | Hypholoma | 0.032 | Undefined Saprotroph |
| cc5bbe01d7b49f4c364863dfaa733ac4 | Agaricomycetes | Baeospora | 0.02 | Undefined Saprotroph |
| 3a2b33bb6a2bf99228e3514fccc73fb6 | Exobasidiomycetes | Tilletiopsis | 0.087 | Endophyte-Litter Saprotroph-Undefined Saprotroph |
| 91a2b4697ae445f4f1c590367115d341 | Microbotryomycetes | Sporobolomyces | 0.024 | Fungal Parasite-Litter Saprotroph |
| 36fd32226e14333d400809aaaa6575c3 | Tremellomycetes | Itersonilia | 0.208 | Litter Saprotroph-Plant Pathogen |
| d2a4e880edf16321f209e6411a0560a8 | Tremellomycetes | Udeniomyces | 0.25 | na |
| 5271e5d43b7a1db5b3297324373abd4b | Tremellomycetes | Hannaella | 0.029 | na |
| 82d7e342b069362f347b318c569a5e33 | Tremellomycetes | Hannaella | 1.556 | na |
| 4bf299390537696958f70c44a1af11ae | Tremellomycetes | Cryptococcus | 0.208 | Animal Pathogen-Endophyte-Epiphyte-Undefined Saprotroph |
| da4ad7f71108b11abccd74e36cb2f452 | Dothideomycetes | Paraphaeosphaeria | 0.157 | Undefined Saprotroph |
| 0d32a5d37e541c527ab7fdf98100fbc2 | Dothideomycetes | Unc. Dothideomycetes | 0.107 | na |
| e40f69f6fcc2a5c9fcb28c4ff08d319c | Dothideomycetes | Unc. Dothideomycetes | 0.044 | na |
| 83996f2a0929abc17e868b2aba781be0 | Leotiomycetes | Unc_Helotiaceae | 0.038 | Ectomycorrhizal-Fungal Parasite-Plant Pathogen-Wood Saprotroph |
| b06d7ec87b48ae65b2fdbea4aa0ef51a | Leotiomycetes | Neobulgaria | 0.105 | Undefined Saprotroph |
| 44b99e8a064c2af80b7d63fd0444bddc | Leotiomycetes | Unc._Helotiales | 1.63 | na |
| 8b1075e33ae08cdfc4be1bd10ea35ca3 | Leotiomycetes | Unc._Helotiales | 0.283 | na |
| a00b66a5981f00f9986e8de72368d72a | Leotiomycetes | Unc._Helotiales | 0.079 | na |
| b9cb830fa2a0517a66cc72da495eebcf | Leotiomycetes | Unc._Helotiales | 0.714 | na |
| bf2b91af110361aa098a82153407d41c | Leotiomycetes | Unc._Helotiales | 2.394 | na |
| c1b786834d4704aaba8de52530748052 | Leotiomycetes | Unc._Helotiales | 0.119 | na |
| e232ae55fc0a9649344f6ec96020bd55 | Pezizomycetes | Peziza | 0.461 | Dung Saprotroph-Ectomycorrhizal-Litter Saprotroph-Undefined Saprotroph |
| 3bd77764cabef86c551d465ca8c63bbc | Sordariomycetes | Plectosphaerella | 1.959 | Endophyte-Plant Pathogen |
| 6e69a6f3ec79d95c17b18266e54d53be | Sordariomycetes | Plectosphaerella | 2.917 | Endophyte-Plant Pathogen |
| 05999798420eb90277e254f067f7ea98 | Sordariomycetes | Sarocladium | 0.211 | Undefined Saprotroph |
| 4809fda2e0604ac3834f12bf7e3f695c | Sordariomycetes | Fusarium | 0.125 | Animal Pathogen-Endophyte-Lichen Parasite-Plant Pathogen-Soil Saprotroph-Wood Saprotroph |
| 806a1f68df44f259e259612aca7ce4ad | Sordariomycetes | Unc._Nectriaceae | 0.032 | Animal Pathogen-Endophyte-Fungal Parasite-Lichen Parasite-Plant Pathogen-Wood Saprotroph |
| 9d8ec7fef0f8d07518c933df849ae298 | Sordariomycetes | Unc._Nectriaceae | 0.026 | Animal Pathogen-Endophyte-Fungal Parasite-Lichen Parasite-Plant Pathogen-Wood Saprotroph |
| bde718847f41be286a85301fdf2cd739 | Sordariomycetes | Unc._Nectriaceae | 0.246 | Animal Pathogen-Endophyte-Fungal Parasite-Lichen Parasite-Plant Pathogen-Wood Saprotroph |
| d1f7226b59e99a9a0b86962eebe128b8 | Sordariomycetes | Unc._Nectriaceae | 0.081 | Animal Pathogen-Endophyte-Fungal Parasite-Lichen Parasite-Plant Pathogen-Wood Saprotroph |
| 1cabb90bfec770a69522ed5501c9204c | Sordariomycetes | Unc._Hypocreales | 0.143 | na |
| 05086b9b0f3caddfaac60d8b249e35ac | Sordariomycetes | Unc. Sordariomycetes | 0.841 | na |
| 3395ebc76e3d50073f92f265d06d7ea0 | Unc. Ascomycota | Unc. Ascomycota | 0.027 | na |
| d257f7577f2dea34b48f6bccacd423dd | Unc. Ascomycota | Unc. Ascomycota | 0.039 | na |
| 8e56264af45e50a57a484d928ab95807 | Agaricomycetes | Psathyrella | 0.061 | Wood Saprotroph |
| 75e64604d0ab18afe068e141e5cb595e | Agaricomycetes | Unc. Auriculariales | 0.313 | na |
| 13751030de438f5e1a86ff24c9834ddc | Agaricomycetes | Unc. Ceratobasidiaceae | 0.083 | Endomycorrhizal-Plant Pathogen-Undefined Saprotroph |
| 317400b92d0452a2deb7a3c95b518739 | Agaricomycetes | Unc. Ceratobasidiaceae | 0.203 | Endomycorrhizal-Plant Pathogen-Undefined Saprotroph |
| 676a05fe9c3bc335998e0dcd7d3798a2 | Agaricomycetes | Unc. Agaricomycetes | 0.048 | na |
| a9b9e65d2db2060061c0acdef5b5ea80 | Agaricostilbomycetes | Kondoa | 0.036 | na |
| 04be918d39b535f6f9b8be516a4557ca | Microbotryomycetes | Leucosporidium | 0.389 | Soil Saprotroph-Undefined Saprotroph |
| 9ad2b19f844b967b49cc51153794b37e | Tremellomycetes | Filobasidium | 0.026 | Undefined Saprotroph |
| dd75ea6d4d79830de79a29b7b4808657 | Tremellomycetes | Solicoccozyma | 0.435 | na |
| 8cc6f570cb70aa02a79a07553a13e935 | Tremellomycetes | Unc. Piskurozymaceae | 0.232 | na |
| 5a1d1c0df7c26548b9bed575ab484bda | Tremellomycetes | Dioszegia | 0.056 | na |
| 73f2936bdd1d96497864bc002c21e6b4 | Tremellomycetes | Dioszegia | 0.039 | na |
| 7538297481157995cb5e32435e0161a2 | Tremellomycetes | Hannaella | 0.94 | na |
| a4149cc0c29db1d1586442c5ef8884e8 | Tremellomycetes | Hannaella | 0.192 | na |
| 2c1c506eed56cfbca61cdd8c174e12ab | Tremellomycetes | Unc. Tremellales | 0.074 | na |
| 4cc334715d4b68bad2f5176d50814406 | Mortierellomycetes | Mortierella | 0.193 | Endophyte-Litter Saprotroph-Soil Saprotroph-Undefined Saprotroph |
| 71eec45193eed470a8a10a148ff03be3 | Mortierellomycetes | Mortierella | 0.169 | Endophyte-Litter Saprotroph-Soil Saprotroph-Undefined Saprotroph |
| 97d864cef61ddec6a59995b4e8170166 | Mortierellomycetes | Mortierella | 0.067 | Endophyte-Litter Saprotroph-Soil Saprotroph-Undefined Saprotroph |
| c89be317eefd2160381cd14252444435 | Mortierellomycetes | Mortierella | 0.437 | Endophyte-Litter Saprotroph-Soil Saprotroph-Undefined Saprotroph |
| cd3cd060909cbaa4504945a56f633999 | Mortierellomycetes | Mortierella | 0.276 | Endophyte-Litter Saprotroph-Soil Saprotroph-Undefined Saprotroph |
| db54f9122156c575c065d56f0c838f38 | Mortierellomycetes | Mortierella | 0.052 | Endophyte-Litter Saprotroph-Soil Saprotroph-Undefined Saprotroph |
| e62a1f870faaa4c71e36d23e08ec7b4d | Mortierellomycetes | Mortierella | 0.047 | Endophyte-Litter Saprotroph-Soil Saprotroph-Undefined Saprotroph |
| b568c1c9d490fbda16eca5f4c50bfc7c | Rozellomycotina_cls_Incertae_sedis | Unc. GS02 | 0.17 | na |
| 1647f941fc2797891326bbd7b0bdfc42 | Leotiomycetes | Unc. Thelebolales | 0.164 | na |
| a4e748c619aa7525d6f16157eacfc4b2 | Agaricomycetes | Minimedusa | 0.086 | na |
| 2c23d7d90e2d9922c0d484c5864335b1 | Microbotryomycetes | Leucosporidium | 2.789 | Soil Saprotroph-Undefined Saprotroph |
| 5610469e3cf08551cb5298e7cfa87c12 | Dothideomycetes | Stagonosporopsis | 5.267 | Plant Pathogen |
| c55bd67c613e3d1a00d8cc70bae61d47 | Dothideomycetes | Unc. Didymosphaeriaceae | 0.071 | Endophyte-Lichen Parasite-Plant Pathogen-Undefined Saprotroph |
| 803e9a7639e21715c3d9d2128758b3c9 | Eurotiomycetes | Cyphellophora | 1.227 | Animal Pathogen-Undefined Saprotroph |
| fe6e0d84335c1c1de1b254c8c3bcd054 | Sordariomycetes | Plectosphaerella | 9.63 | Endophyte-Plant Pathogen |
| eee66ab32802b2911297e77f9bd8e2a6 | Unc. Ascomycota | Unc. Ascomycota | 2.436 | na |
| cbbb38bd7df5194437c9f2d0475c7c4d | Agaricomycetes | Unc. Ceratobasidiaceae | 0.067 | Endomycorrhizal-Plant Pathogen-Undefined Saprotroph |

**Supplementary Table S4.** Bacterial identification based on partial 16S rRNA genes of bacteria isolated from Riccia

| Isolate ID | Closest match | Similarity (%) | Putative role | Reference |
| --- | --- | --- | --- | --- |
| RIC_8 | *Pseudomonas alkylphenolica* | 98.31% | alkylphenol degradation | Song, M. M., Veeranagouda, Y., Ganzorig, M., & Lee, K. (2018). Circular pellicles formed by Pseudomonas alkylphenolica KL28 are a sophisticated architecture principally designed by matrix substance. Journal of Microbiology, 56(11), 790-797. |
| RIC_11 | *Pseudomonas alkylphenolica* | 99.44% |  |  |
| RIC_12 | *Pseudomonas alkylphenolica* | 99.71% |  |  |
| RIC_14 | *Pseudomonas asplenii* | 99.62% | a causal agent of bacterial soft rot disease in cyclamen plant and a bacterial pathogen in Birds nest fern (*Asplenium nidus*) | 1) Rodríguez-Parra, J. A., Moreno-López, J. P., & González-Almario, A. (2022). Dickeya solani, Pectobacterium atrosepticum and Pseudomonas asplenii: causal agents of bacterial soft rot in cyclamen plants (Cyclamen persicum Mill.) in Colombia. Canadian Journal of Plant Pathology, 1-19. 2) Ude, S., Arnold, D. L., Moon, C. D., Timms‐Wilson, T., & Spiers, A. J. (2006). Biofilm formation and cellulose expression among diverse environmental Pseudomonas isolates. Environmental microbiology, 8(11), 1997-2011. |
| RIC_16 | *Pseudomonas furukawaii* | 99.53% | polychlorinated biphenyl-degradation | Kimura, N., Watanabe, T., Suenaga, H., Fujihara, H., Futagami, T., Goto, M., ... & Hirose, J. (2018). Pseudomonas furukawaii sp. nov., a polychlorinated biphenyl-degrading bacterium isolated from biphenyl-contaminated soil in Japan. International journal of systematic and evolutionary microbiology, 68(5), 1429-1435. |
| RIC_23 | *Pseudomonas* *laurentiana* | 99.35% | antagonism against fungal pathogens | Rafikova, G. F., Kuzina, E. V., Korshunova, T. Y., & Loginov, O. N. (2020). New bacterial strains of Рseudomonas laurentiana: promising agents for agrobiotechnology. Moscow University Biological Sciences Bulletin, 75(4), 206-211. |
| RIC_33 | *Pseudomonas laurentiana* | 99.81% |  |  |
| RIC_9 | *Pseudomonas putida* | 97.67% | plant growth-promotion i.e., salt stress tolerance, antagonism against fungal pathogens and phytohormone production | 1) Costa-Gutierrez, S. B., Lami, M. J., Santo, M. C. C. D., Zenoff, A. M., Vincent, P. A., Molina-Henares, M. A., ... & de Cristóbal, R. E. (2020). Plant growth promotion by Pseudomonas putida KT2440 under saline stress: role of eptA. Applied microbiology and biotechnology, 104(10), 4577-4592. 2) Berg, G., Krechel, A., Ditz, M., Sikora, R. A., Ulrich, A., & Hallmann, J. (2005). Endophytic and ectophytic potato-associated bacterial communities differ in structure and antagonistic function against plant pathogenic fungi. FEMS Microbiology Ecology, 51(2), 215-229. |
| RIC_18 | *Pseudomonas putida* | 99.91% |  |  |
| RIC_19 | *Pseudomonas putida* | 99.52% |  |  |
| RIC_22 | *Pseudomonas putida* | 98.40% |  |  |
| RIC_25 | *Pseudomonas putida* | 99.90% |  |  |
| RIC_27 | *Pseudomonas putida* | 99.56% |  |  |
| RIC_32 | *Pseudomonas putida* | 99.64% |  |  |
| RIC_13 | *Pseudomonas resinovorans* | 99.62% | phenol-degradation | 1) Yang, C. F., & Lee, C. M. (2007). Enrichment, isolation, and characterization of phenol-degrading Pseudomonas resinovorans strain P-1 and Brevibacillus sp. strain P-6. International Biodeterioration & Biodegradation, 59(3), 206-210. |
| RIC_20 | *Pseudomonas resinovorans* | 99.62% |  |  |
| RIC_21 | *Pseudomonas* *psychrotolerans* | 99.54% | plant growth-promotion i.e., heavy metal tolerance, fixing nitrogen and salt stress tolerance | 1) Kang, S. M., Asaf, S., Khan, A. L., Khan, A., Mun, B. G., Khan, M. A., ... & Lee, I. J. (2020). Complete genome sequence of Pseudomonas psychrotolerans CS51, a plant growth-promoting bacterium, under heavy metal stress conditions. Microorganisms, 8(3), 382. 2) Kubi, H. A. A., Khan, M. A., Adhikari, A., Imran, M., Kang, S. M., Hamayun, M., & Lee, I. J. (2021). Silicon and plant growth-promoting Rhizobacteria Pseudomonas psychrotolerans CS51 mitigates salt stress in Zea mays L. Agriculture, 11(3), 272. 3) Liu, R., Zhang, Y., Chen, P., Lin, H., Ye, G., Wang, Z., ... & Ren, D. (2017). Genomic and phenotypic analyses of Pseudomonas psychrotolerans PRS08-11306 reveal a turnerbactin biosynthesis gene cluster that contributes to nitrogen fixation. Journal of biotechnology, 253, 10-13. |
| RIC_3 | *Xanthomonas* *translucens* | 99.79% | a causal agent of bacterial leaf streak in of wheat and cereal crop | 1. Sapkota, S., Mergoum, M., & Liu, Z. (2020). The translucens group of Xanthomonas translucens: Complicated and important pathogens causing bacterial leaf streak on cereals. Molecular plant pathology, 21(3), 291-302. |
| RIC_24 | *Xanthomonas translucens* | 99.71% |  |  |
| RIC_35 | *Xanthomonas translucens* | 98.89% |  |  |
| RIC_36 | *Xanthomonas translucens* | 99.49% |  |  |
| RIC_39 | *Xanthomonas translucens* | 96.54% |  |  |
| RIC_38 | *Xanthomonas translucens* | 100.00% |  |  |
| RIC_10 | *Xanthomonas translucens* | 98.92% |  |  |
| RIC_15 | *Xanthomonas translucens* | 99.81% |  |  |
| RIC_24 | *Xanthomonas translucens* | 99.72% |  |  |
| RIC_28 | *Xanthomonas translucens* | 99.90% |  |  |
| RIC_30 | *Xanthomonas translucens* | 99.81% |  |  |
| RIC_37 | *Xanthomonas translucens* | 99.11% |  |  |
| RIC_1 | *Xanthomonas translucens* pv*. undulosa* | 99.09% |  |  |

**
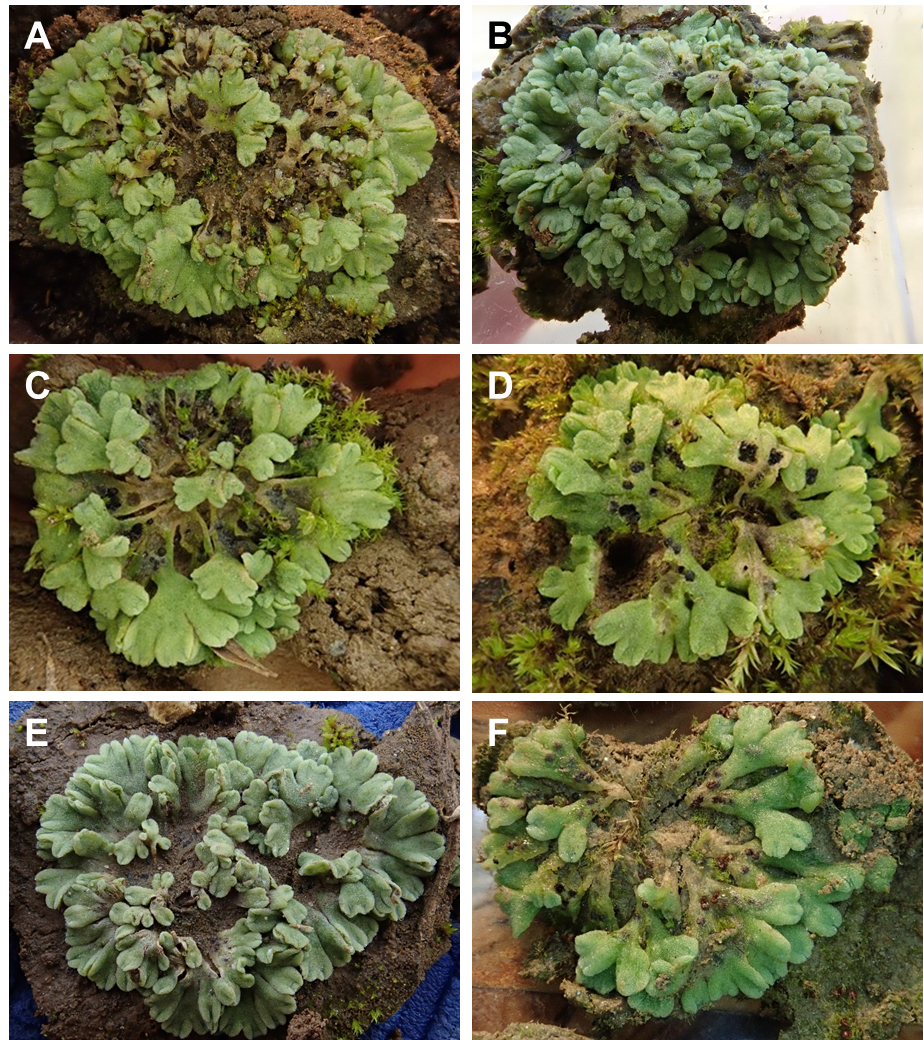
**

**Supplementary Figure S1.** Photographs of selected *Riccia* samples that were analyzed as part of this study. A-C and E-F – *Riccia bifurca*; D – *Riccia glauca*.


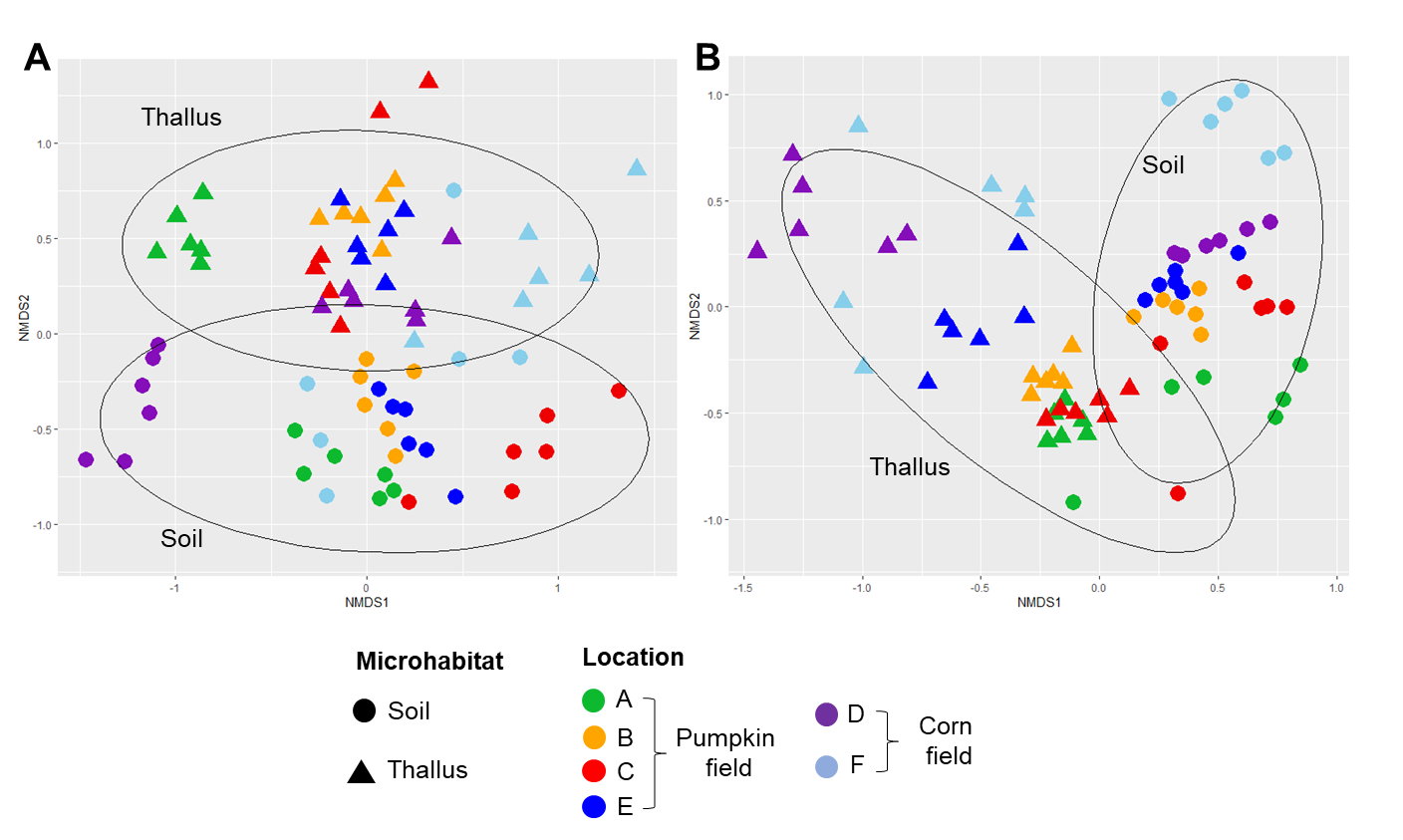


**Supplementary Figure S2.** NMDS plots showing clustering of bacterial (A) and fungal (B) community structures in thallus and soil samples.


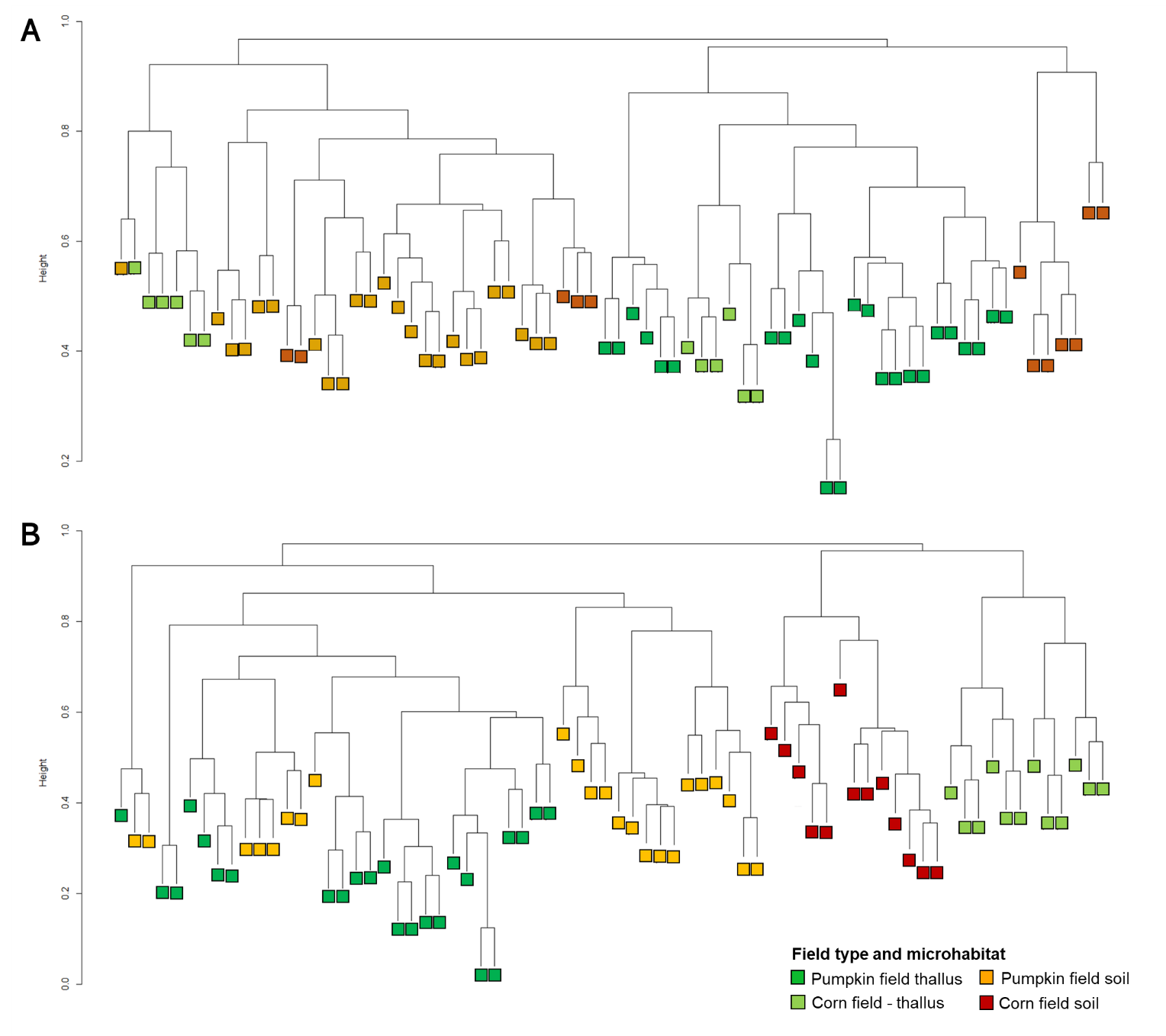
**Supplementary Figure S3.** Hierarchical clustering of bacterial (A) and fungal (B) community structures of thallus and soil samples that were obtained from different field types.


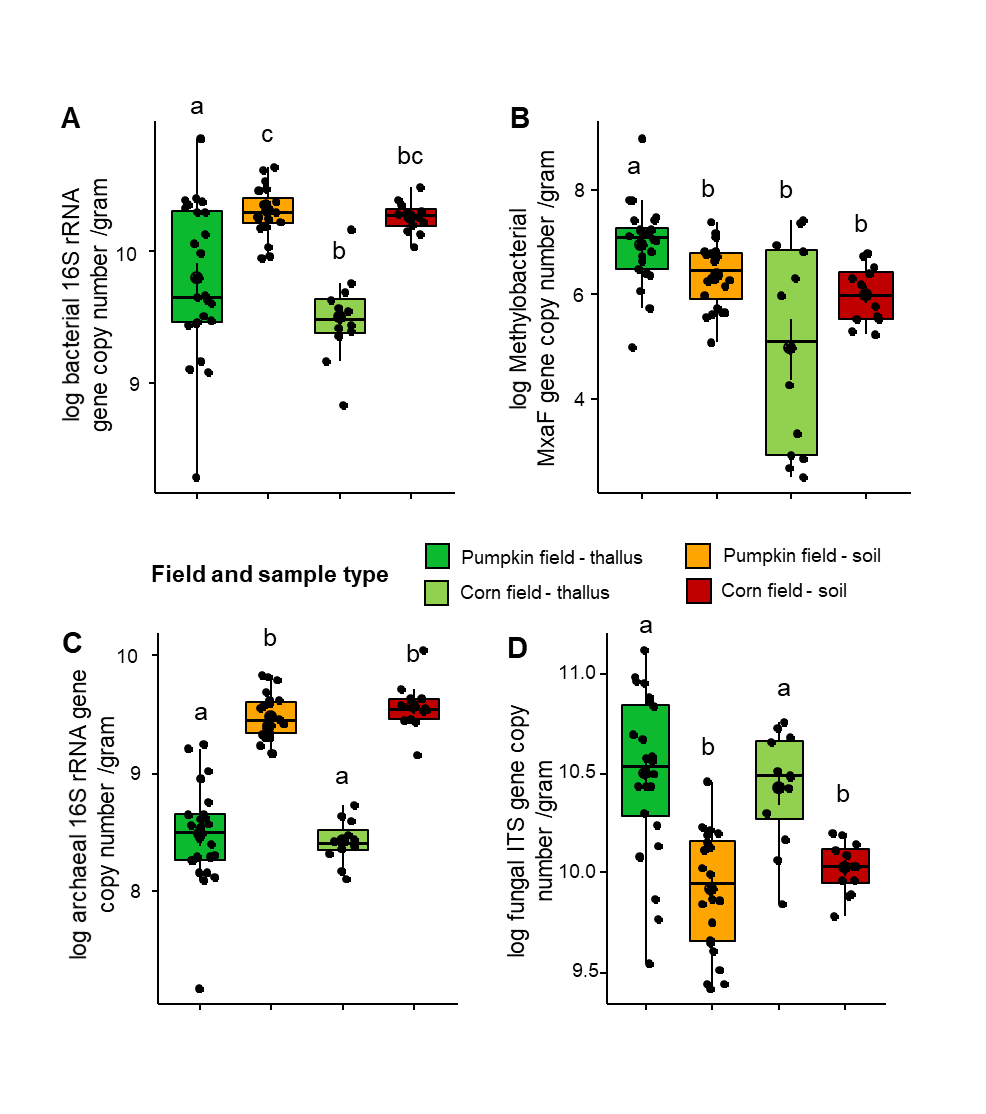


**Supplementary Figure** **S4**. **Comparisons of microbial abundances between soil and liverwort thalli that were collected from different field types.** Bacterial (A) and methylobacterial (B), abundances were quantified using a qPCR-based approach and transformed into log values. Data are presented as the mean for each group. The different letters above each bar indicate statistical significance at P_adj_ < 0.05 based on post-hoc Dunn’s test for multiple pairwise comparisons.


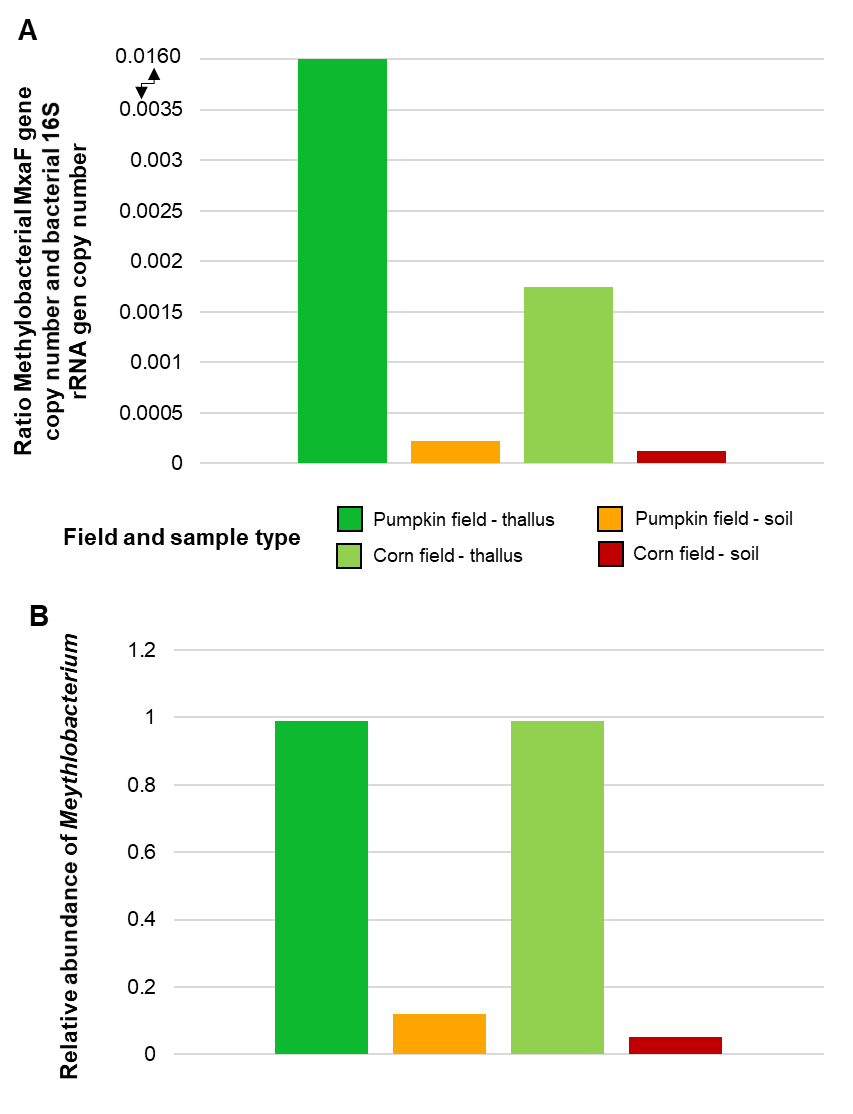


**Supplementary Figure S5.** Ratio between the methylobacterial *MxaF* gene copy number and bacterial 16S rRNA gene copy number based on qPCR analysis (A) and relative abundance of the genus *Methylobacterium* in thallus and soil samples from different fields based on amplicon sequencing (B).

**
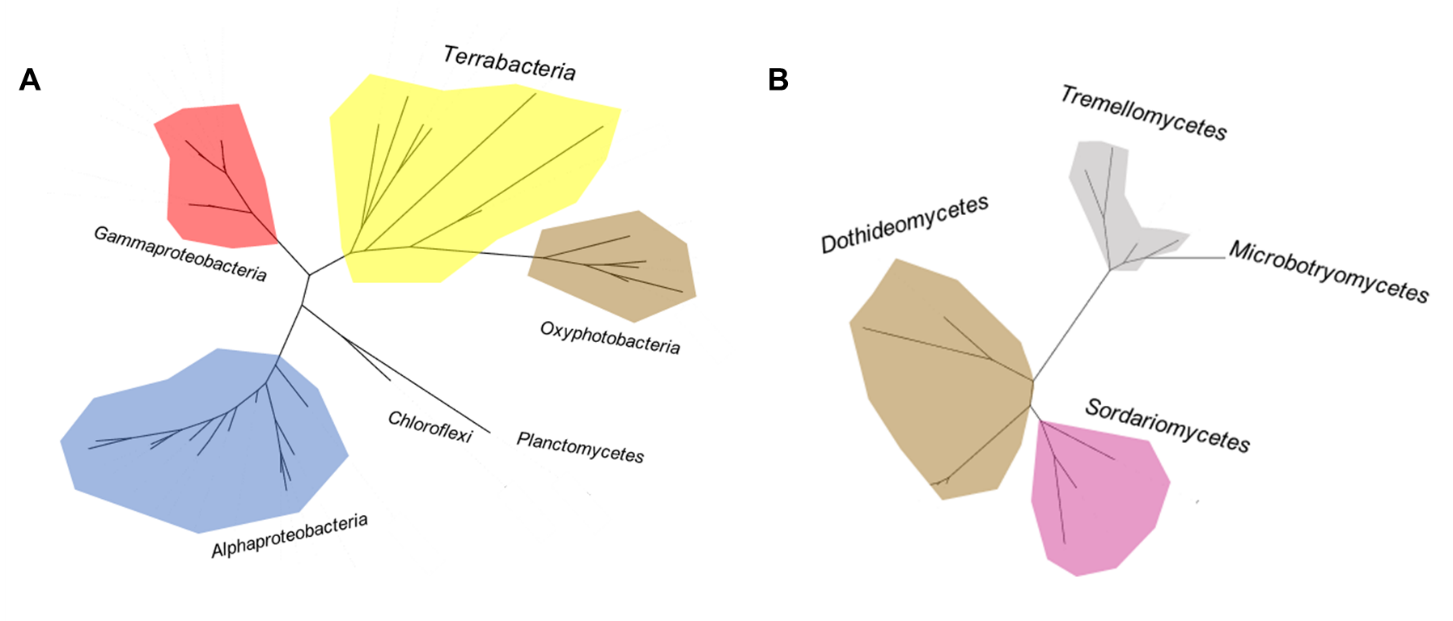
**

**Supplementary Figure S6. Core microbiome assessment.** The core microbiome of prokaryotic (A) and fungal (B) communities contains only ASVs that were present in at least 90% of the total samples. **A phylogenetic tree was constructed with partial 16S rRNA gene and ITS region sequences of the core microbiome (A:** prokaryotes **and B: fungi).**


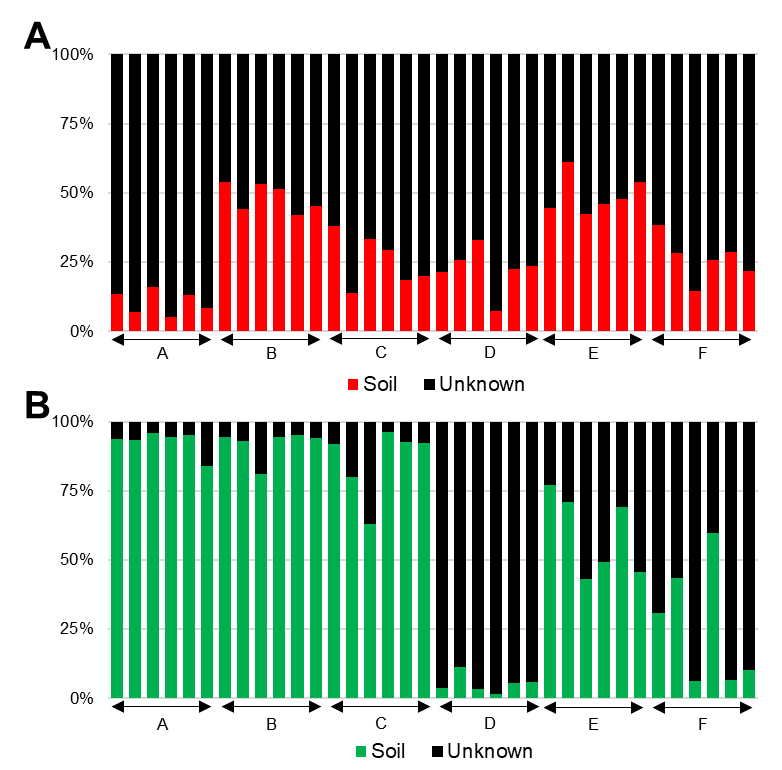


**Supplementary Figure S7.** Tracking of bacterial (A) and fungal (B) microbiome constituents in thallus samples from surrounding soil samples. The percentages are based on SourceTracker2 analysis in R.
